# Recent Advances in Brillouin Optical Time Domain Reflectometry

**DOI:** 10.3390/s19081862

**Published:** 2019-04-18

**Authors:** Qing Bai, Qinglin Wang, Dong Wang, Yu Wang, Yan Gao, Hongjuan Zhang, Mingjiang Zhang, Baoquan Jin

**Affiliations:** 1 Key Laboratory of Advanced Transducers and Intelligent Control Systems (Ministry of Education and Shanxi Province), Taiyuan University of Technology, Taiyuan 030024, China; baiqing0122@link.tyut.edu.cn (Q.B.); wangqinglin0145@link.tyut.edu.cn (Q.W.); wangdong@tyut.edu.cn (D.W.); wangyu@tyut.edu.cn (Y.W.); gaoyan@tyut.edu.cn (Y.G.); zhanghongjuan@tyut.edu.cn (H.Z.); 2 College of Physics and Optoelectronics, Taiyuan University of Technology, Taiyuan 030024, China; zhangmingjiang@tyut.edu.cn; 3 State Key Laboratory of Coal and CBM Co-mining, Shanxi Jincheng Anthracite Mine Group Co., Ltd., Jincheng 048000, China

**Keywords:** distributed optical fiber sensing, BOTDR, spatial resolution, signal-to-noise ratio, cross-sensitivity, novel fiber, BOTDR application

## Abstract

In the past two decades Brillouin-based sensors have emerged as a newly-developed optical fiber sensing technology for distributed temperature and strain measurements. Among these, the Brillouin optical time domain reflectometer (BOTDR) has attracted more and more research attention, because of its exclusive advantages, including single-end access, simple system architecture, easy implementation and widespread field applications. It is realized mainly by injecting optical pulses into the fiber and detecting the Brillouin frequency shift (BFS), which is linearly related to the change of ambient temperature and axial strain of the sensing fiber. In this paper, the authors provide a review of new progress on performance improvement and applications of BOTDR in the last decade. Firstly, the recent advances in improving the performance of BOTDRs are summarized, such as spatial resolution, signal-to-noise ratio and measurement accuracy, measurement speed, cross sensitivity and other properties. Moreover, novel-type optical fibers bring new characteristics to optic fiber sensors, hence we introduce the different Brillouin sensing features of special fibers, mainly covering the plastic optical fiber, photonic crystal fiber, few-mode fiber and other special fibers. Additionally, we present a brief overview of BOTDR application scenarios in many industrial fields and intelligent perception, including structural health monitoring of large-range infrastructure, geological disaster prewarning and other applications. To conclude, we discuss several challenges and prospects in the future development of BOTDRs.

## 1. Introduction

Since extremely-low-loss glass optical fiber was first manufactured in 1970s [1], it has been treated as the optimal information transmission carrier for high-speed and super-capacity data communication systems. Subsequently, after deeper investigation into the properties of optical fibers, researchers gradually found that optical fiber possesses inherent advantages in long-range distributed optical fiber sensing (DOFS) due to its capability of simultaneously acting as the physical sensing element and the signal transmission medium. Hence, an ever-increasing number of optical fiber sensors were developed by analyzing different optical effects of optical fiber, such as Rayleigh, Raman and Brillouin backscattering [2,3] or FBG spectra [4].

The Rayleigh backscattering effect was originally utilized in an optical time domain reflectometer (OTDR) for evaluating the attenuation and imperfections of the optical fiber [5], and further utilized in a polarization-OTDR (POTDR), a coherent OTDR (COTDR) and a phase-sensitive OTDR (ϕ-OTDR) for vibration measurement [6,7]. Raman backscattering was related with the temperature change and was utilized in a class of distributed temperature sensors named Raman OTDRs since 1989 [8]. Brillouin backscattering is sensitive to both the temperature and strain, attributed to a linear relationship of the Brillouin frequency shift (BFS) versus the temperature and strain change [9,10]. Various types of Brillouin-based sensors were developed since the 1990s for both temperature and strain measurement, such as the Brillouin optical time domain analysis (BOTDA) [11] and reflectometer (BOTDR) [12], Brillouin optical frequency domain analysis (BOFDA) [13] and reflectometer (BOFDR) [14], Brillouin optical correlation domain analysis (BOCDA) [15] and reflectometer (BOCDR) [16], and Brillouin dynamic grating distributed sensing [17,18].

Among the Brillouin-based sensors, the Brillouin analysis techniques (BOTDA, BOFDA, BOCDA) are based on the Brillouin interaction between pump pulse and probe continuous light waves simultaneously counterpropagating in optical fibers. These techniques inevitably suffer from the disadvantage that both ends of the sensing fiber have to be accessed, which limits their application in some engineering fields. Though the BOCDR and BOFDR offer the advantage of one-end access, their largest sensing distance was only around several kilometers. Compared with the techniques mentioned above, the BOTDR features the superiority of both single-end access and long sensing distance (several tens of or more than 100 kilometers), and has been widely utilized in structural health monitoring of large infrastructures [19,20]. Hence, the BOTDR has drawn much research interest in the last decade driven by the application requirement, and lots of schemes were proposed for its performance enhancement, mainly including the spatial resolution (SR), signal to noise ratio (SNR) determining the sensing range, measurement accuracy and measurement speed.

The SR is an important issue in a sensor because high SR ensures the capacity of refined spatial measurement. For instance, sub-centimeter-scale or millimeter-scale SR is always necessary for biomedical measurement and diagnosis [21,22]. In Brillouin sensors, it has been demonstrated that the detected BFS value will deviate from the actual one if the temperature or strain change occurs over a length of the fiber shorter than the SR [23]. For Brillouin analysis techniques, cm-scale or even mm-scale SR has been achieved [15,24,25,26], while sub-meter SR for BOTDR is relatively hard to achieve. Therefore, researchers have proposed various methods to improve the SR of BOTDR, as listed below in Section 2.2.

It has been verified that the BOTDR inherently suffers from relatively-low SNR in principle, because the probe light is unidirectionally injected and only low-intensity spontaneous Brillouin scattering (SpBS) is detected. High SNR signifies the high measurement accuracy and the large measurement range for BOTDR. In terms of enhancing the SNR and measurement accuracy, many improved schemes were proposed based on hardware optimization, such as seed laser performance, probe pulse features and optical link structures. Besides, various demodulation algorithms were also proposed, as summarized in Section 2.3.

In conventional BOTDR, the Brillouin gain spectrum (BGS) is generally obtained through frequency-scanning and curve-fitting method. Due to the low SNR, a huge number of signal averagings (more than 2^13^) is essential for acquiring the power trace at every frequency point. This process of frequency scanning and signal averaging is so time-consuming that the measurement speed is limited severely. Hence, researchers have proposed various optimized algorithms for accelerating the signal measurement and curve fitting. Besides, several new principles and implementations without the request of frequency scanning are also demonstrated in BOTDR for faster speed, as concluded in Section 2.4.

Since the BFS is linearly proportional to both the temperature and strain at the same time, it’s theoretically impossible to separate these two effects by only measuring one BFS. This cross-sensitivity problem limits the practical use of BOTDR systems. An early solution was to add another fiber isolated from the strain effects to monitor temperature only to modify the BFS [27]. Then, both the BFS and the Brillouin power level were simultaneously investigated to discriminate the temperature and strain [28,29]. In the past ten years, several hybrid sensing mechanisms and setups, such as the combination of BOTDR and ROTDR, have been proposed aiming at removing the cross-talk of temperature and strain in BOTDR, as demonstrated in Section 2.5. Additionally, some other performance features of BOTDR have also attracted a lot of attention, as shown in Section 2.6.

Recently, various new types of fibers have gradually displayed better performance and lower cost with the improvement of fiber fabrication technology, which brings about new performances and chances for Brillouin sensors. For example, plastic optical fibers can overcome shortcomings of conventional optical fibers such as their fragility and be more suitable for large strain detection, as reviewed in Section 3.1. The photonic-crystal fiber and few-mode fiber features of multi-peak BGSs, which provide available methods of distinguishing temperature and strain utilizing only one fiber, as described in Section 3.2 and Section 3.3. Besides, other special fibers also have potential for solving the temperature and strain cross-sensitivity issue in BOTDRs as listed in Section 3.4, such as large effective-area fiber, erbium-doped fiber and multi-core fiber.

In this paper, the recent advances of BOTDRs in the last decade are reviewed. Their performance enhancement, including the spatial resolution (SR), signal to noise ratio (SNR) and measurement accuracy, measurement speed and cross-sensitivity, is summarized in Section 2. Then, the new Brillouin scattering features of several-type novel fibers are summarized in Section 3, which provides new methods for performance enhancement of Brillouin sensing. In Section 4, the BOTDR applications are listed briefly, mainly in the fields of structural health monitoring (SHM) and geological disaster prewarning. As a conclusion, we discuss several challenges and prospects in the future development of BOTDRs.

## 2. Performance Improvement of BOTDRs

In the last decade, a variety of schemes have been put forward to improve the main performance features of BOTDRs. Besides, other issues in BOTDR have also attracted the attention of researchers, such as the cross-sensitivity of temperature and strain, location error, electronic-bandwidth reduction, system structure simplification and multi-parameter integrated sensing. In this section, we firstly introduced the spontaneous Brillouin scattering (SpBS) and further present the principle of BOTDR in brief, and then expanded details with emphasis on BOTDR performance improvements.

### 2.1. Principle of BOTDR

**Figure 1:**
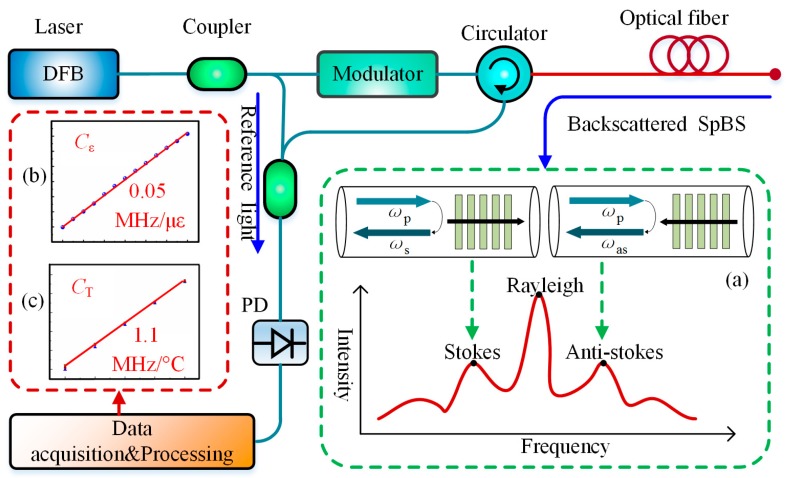
Basic structure of BOTDR: (**a**) Stokes and Anti-Stokes light; (**b**) linear relationship between BFS and strain; (**c**) linear relationship between BFS and temperature.

The basic structure of a BOTDR sensing system is shown in Figure 1. The continuous light from the laser is modulated into pulsed light by a modulator and injected into the sensing fiber to generate the SpBS. By detecting the power or BFS of SpBS, the temperature or strain along the fiber can be determined.

The SpBS is a type of inelastic scattering caused by the interaction between the incident light field and the acoustic phonons inside the optical fiber. Because of the random thermal motion of material particles in optical fiber, a spontaneous acoustic wave forms and can be viewed as a grating moving forward or backward with a certain velocity along the fiber. When the probe light with the optical frequency of *ω*_p_ is injected into the fiber, the probe light interacts with the moving acoustic gating (MAG), which leads to the optical frequency shift of probe light due to the Doppler effect and the spontaneous formation of Brillouin scattering, as shown in Figure 1a. When the probe light propagates in the same direction as the MAG, a light wave with a lower frequency of *ω*_s_ is generated, named as the Stokes light; when the probe light propagates in the opposite direction as the MAG, the light wave with a higher frequency of *ω*_as_ is generated named as the Anti-Stokes light.

The frequency shift between the incident light and the Stokes or Anti-Stokes light is called Brillouin frequency shift (BFS), which can be expressed as Equation (1) [10]:

(1) $$v_{B}=\omega_{\mathrm{as}}-\omega_{p}=\omega_{p}-\omega_{s}=\frac{2nV}{\lambda }$$

where *n* is the refractive index of the optical fiber, *V* is the acoustic speed in fiber, *λ* is the wavelength of the probe light. For the single mode quartz optical fiber in room temperature (*T* = 20 °C), the BFS is about 11 GHz with a pump wavelength of 1550 nm. When the temperature and axial strain of the fiber is changed, the BFS will change, following the Equation (2):

(2) $$v_{B}\left(T,\varepsilon \right)\approx v_{B}\left(T_{0},\varepsilon_{0}\right)+C_{T}(T-T_{0})+C_{\varepsilon }(\varepsilon -\varepsilon_{0})$$

where *T*_0_ and *ε*_0_ is the initial temperature and strain, respectively, *C*_T_ and *C_ε_* are the temperature and strain coefficient of BFS, respectively (approximately 1.1 MHz/°C and 0.05 MHz/με [28]). The location *L* where the BFS changes can be calculated according to Equation (3):

(3) $$L=\frac{ct}{2n}$$

where *c* is the light speed in vacuum, *t* is the flight time of probe pulse.

### 2.2. Spatial Resolution(SR)

The measurement result of BOTDR is proverbially inaccurate when the length of a sensing fiber segment is shorter than the spatial resolution. Traditionally, it is accepted that the spatial resolution (SR) is no better than 1 m for conventional Brillouin time-domain sensors due to the limitation of phonon relaxation time [24,30] and the trade-off between SR and BFS resolution when measuring with a conventional BOTDR system [31], for the reason that the measured Brillouin gain spectrum (BGS) broadens sharply when the probe pulse narrows below 10 ns, which brings increasing BFS error. However, 1 m SR is always not enough at special situations such as the health monitoring of fine structures, and the SR is related closely not only to the pulse width but also many system parameters [18,32]. Hence, much attention has been paid to improving the SR, and several novel techniques have been proposed in the recent decade.

**Figure 2:**
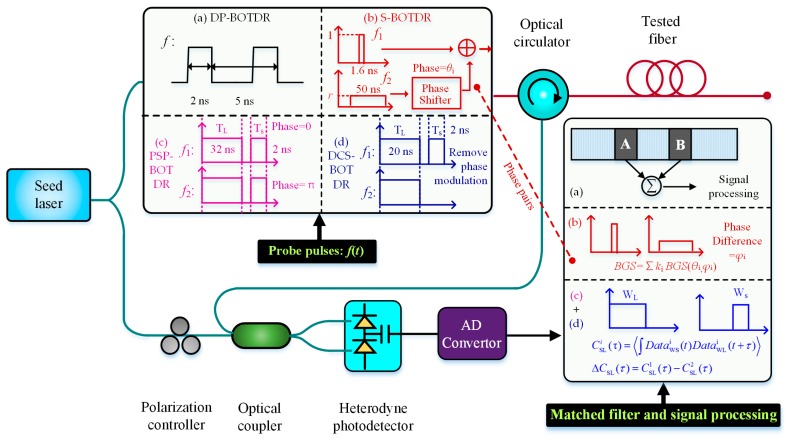
Summarized block diagrams of the four mentioned high-SR BOTDRs: (**a**) DP-BOTDR; (**b**) S-BOTDR; (**c**) PSP-BOTDR; (**d**) DCS-BOTDR.

Researchers found that the coherence characteristics of spontaneous Brillouin scattering originating from two adjacent narrow pulses can be utilized for improving the SR of BOTDR [33]. Based on this principle, a series of new-type BOTDRs were developed one after another, including a double-pulse BOTDR (DP-BOTDR), a synthetic BOTDR (S-BOTDR), a phase shift pulse BOTDR (PSP-BOTDR) and a differential cross spectrum BOTDR (DCS-BOTDR). Figure 2 simply summarizes the block diagrams of four mentioned high-SR BOTDRs, including probe pulses, matched filters and signal processing.

In 2007, Koyamada et al. realized the DP-BOTDR by injecting two probe pulses into the sensing fiber with the pulse width of 2 ns and the front-pulse to rear-pulse time interval of 5 ns. This DP-BOTDR system enabled them to measure BFS distribution with a sub-meter spatial resolution of 20 cm [34,35], which is five times better than that provided by the conventional single-pulse BOTDR system. In 2014, Nishiguchi et al. proposed the S-BOTDR, the basic principle of which is constructing a two-dimensional δ-like-function both in time domain and frequency domain for improving the spatial and frequency resolution simultaneously [36]. In S-BOTDR, a short pulse and a long pulse were injected into the optical fiber with the phases difference of *θ* and the amplitude ratio of *r*, then the Brillouin scattering signals caused by the two pulses were collected, respectively, by using two matched low-pass filter also with a phase difference of *ϕ*. That is, four BOTDR measurements utilizing four types of probe pulses were performed in order using four given pairs of (*θ*, *ϕ*), and the final BGS was calculated by synthesizing the four BGS with specific weights. The experimental results showed that a 10 cm SR over 40 m fiber was achieved. In 2016, Shibata et al. proposed the PSP-BOTDR [33,37], the probe pulses of which consist of long and short pulses modulated by phase shift keying. The high SR was realized based on the cross-correlation analysis of backscattered Brillouin signals, which are sampled with two matched rectangular window functions with different time lengths according to the probe pulses width. A SR of 0.2 m with a frequency accuracy of 1.08 MHz was achieved over a 354.4 m tested fiber. Then, for easy handing, they recently removed the phase modulation and used only intensity modulation to generate a pair of pulsed probes, which means that the first probe was composed of long and short pulses while the second just the long pulse. The SR of 0.2 m over 354.4 m was ensured utilizing the differential cross spectrum technique, which can be called as the DCS-BOTDR [38].

**Figure 3:**
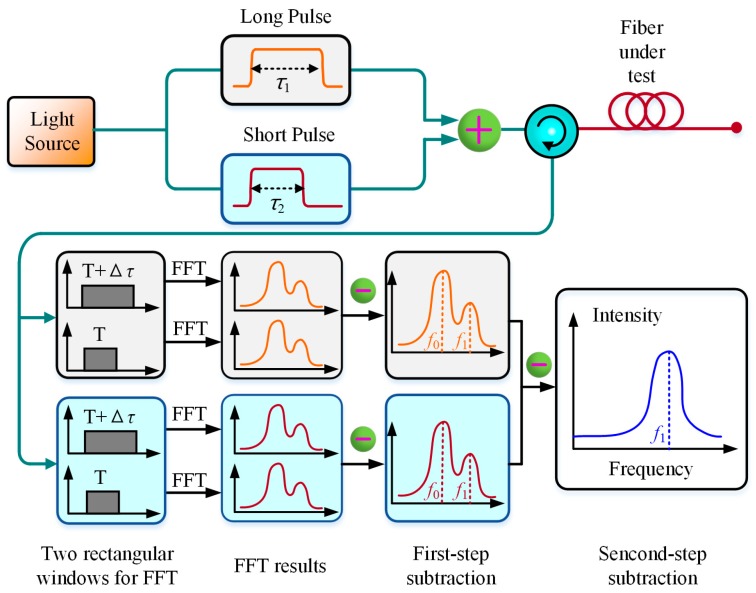
Differential-pulse-pair technique based on two-step subtraction.

The differential-pulse-pair technique has been reported to achieve cm-scale SR in BOTDA [24,25]. Similarly, it can be also utilized in BOTDR for higher SR. In 2016, a pulse pair with slight width difference was employed as probe light to achieve high-SR BOTDR, where a two-step differential Brillouin spectrum technique was also adopted to ensure sub-meter spatial resolution [39]. Different from [36], the SR in [39] was determined mainly by the width difference of the pulse pair not the shorter pulse width, where 0.4 m SR is realized over 7.8 km sensing length by utilizing 60/56 ns pulse pair. In 2018, Yu et al. likewise achieved a spatial resolution of 0.2 m over a 3 km sensing fiber based on the differential-pulse-pair technique, then further discussed the influence of different pulse-width difference and different rise/fall time on the SR of BOTDR [40]. The differential-pulse-pair technique based on two-step subtraction is presented in Figure 3.

**Figure 4:**
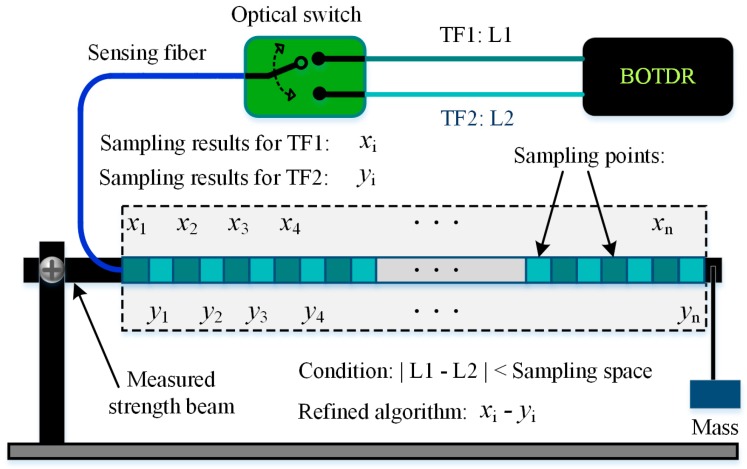
Schematic diagram of the equidistance difference optimum method.

In field application, a practical technique named the equidistance difference optimum method was proposed by He et al. in 2013, which was capable of eliminating the distortion of the strain information arising from the mismatched caused by the measured fiber length being shorter than the SR during the temperature and strain measurement by BOTDR [41]. In this technique, two transmission fibers (TF1 and TF2) with different lengths (L1 and L2) were connected to the BOTDR via an optical switch. Then the strain distribution can be refined by the difference data between the two measured results obtained by using F1 and F2 as transmission lines to acquire the Brillouin scattering signal with the same sampling interval. The technique could detect the damage more effectively including any point of the measuring zone. Its basic schematic diagram is shown in Figure 4.

**Figure 5:**
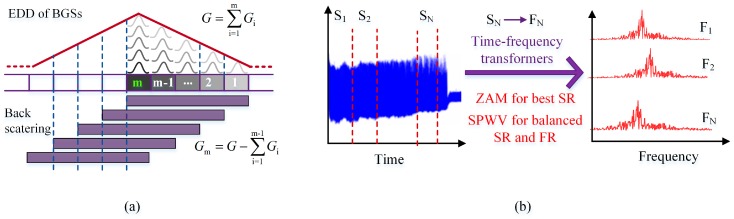
Signal processing methods for high-SR BOTDR: (**a**) iterative subdivision of BGSs; (**b**) optimized time-frequency transformers.

In addition to the hardware optimizations mentioned above, several signal processing methods have been carried out to improve the spatial resolution. In 2013, Wang et al. put forward an iterative subdivision method to improve the spatial resolution of BOTDR. They derived the energy density distribution (EDD) of BGSs by dividing the sensing fiber into equal-length segments with sub-SR length and allowing for both the effect of the probe pulse width and the system response time. Then the Brillouin signals generated from the sub-SR–long fiber segments were extracted through the iterative subdivision. The SR of 0.1 m over a 1 km sensing fiber with a probe pulse of 10 ns and a SR of 1.5 m in a 50 km sensing fiber with a probe pulse of 100 ns were achieved [42]. Similarly, Zhang et al. proposed a pulse subdivision superposition (PSS) method to improve the SR in 2017 [43]. Unlike the former, they omitted the EDD analysis and treated the measured BGS as the superposition of sub-BGSs generated by sub-SR-length probe pulses within the given SR. The high-SR measurement was realized by simply acquiring signals with a high rate and subtracting the sub-BGSs of the neighboring data points. The SR of 0.1 m with a probe pulse of 10 ns was obtained by this method. In 2017, Yu et al. compared the influences of multiple time-frequency transformers on the spatial and frequency resolution of BOTDR [44], which indicated that the Zhao-Atlas-Marks (ZAM) transform brings the best spatial resolution and the smoothed pseudo Wigner–Ville transform (SPWV) can provide better resolution in both the distance domain and the frequency domain. Figure 5 gives the signal processing methods for high-SR BOTDR.

Additionally, Feng et al. in 2013 proposed a novel stationary wavelet-transform (SWT) approach for multi-resolution analysis (MRA) [45]. Different from the aforementioned BGS processing methods, this one was operated based on the already-obtained strain-time distribution data by BOTDR. Through further decomposing the obtained data and extracting the approximate coefficients of the strain data, the system noise and Brillouin frequency-peak shift distortions would be eliminated. Hence, the SNR could be improved significantly and the strain peak on the region less than the resolution of BOTDR can be detected. By using the proposed postprocessing approach, they detected joint opening displacements below the 50 μm level with a common BOTDR with 1 m resolution.

### 2.3. SNR and Measurement Accuracy

As mentioned before, the BOTDR has the advantage of one-end access. However, it is intrinsically subjected to the lower SNR compared with the Brillouin analysis technique, because only the probe pulse light (no pump continuous light) propagates in the fiber. It is well known that the higher SNR means the large sensing range for BOTDR. Meanwhile, the measurement error will decrease as the SNR increase according to Equation (1):

(4) $$\Delta \alpha =\frac{W_{\mathrm{BGS}}}{\sqrt{2}{\left(SNR\right)}^{1/4}}$$

where *W*_BGS_ is the 3dB-linewidth of the BGS.

Hence, how to improve the SNR of BOTDR for larger sensing distance and smaller measurement error is always a research hotspot. In this section, we summarize four types of schemes to improve the SNR of BOTDR, which are respectively related with the seed laser performance (laser linewidth and wavelength), the features of probe pulses, the structure of the optical link, and demodulation algorithms.

#### 2.3.1. Optimization of Seed Laser Performance

As an important parameter of the laser, the linewidth imposes a broadening effect on BGS, which affects the SNR to some degree. In 2013, Hao et al. investigated the influences of different laser linewidths on the BOTDR sensing system. The results showed that as the linewidth exceeded 1 MHz, the measured BGS was broadened severely and the SNR was degraded, but the broadening effect is indistinctive when the laser linewidth is less than 1 MHz [46]. Recently, Bai et al. further numerically simulated and verified experimentally the influence of laser linewidth on the SNR and the BFS measurement accuracy when it is less than 1 MHz. The experimental results indicated that the BFS accuracy improves with the laser linewidth narrowing. However, the BFS accuracy will deteriorate when the laser linewidth decreases to a certain value (reported as 98 Hz in [47]), which may arise from the increasing coherent Rayleigh noise (CRN) related closely with the narrowing linewidth. The above reports will be helpful to choose an appropriate laser for BOTDR.

Besides, the laser wavelength was related to the SNR of BOTDR and the wavelength diversity technique (WDT) benefited the SNR enhancement of BOTDR. In 2012, Li et al. conducted a BOTDR experiment based on multi-wavelength coherent detection, which was realized by injecting the probe pulse light with different wavelengths and detecting the overlapped multiple BGS arising from the each probe wavelength. By using three probe wavelengths in BOTDR, 4.2 dB SNR improvement (SNRI) was obtained and the measurement accuracy was improved twice at the end of 23.6 km fiber compared with the conventional single-wavelength BOTDR [48].

**Figure 6:**
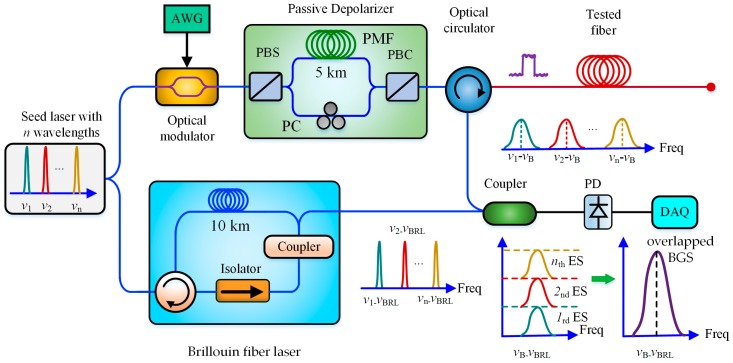
Schematic block diagram of multi-wavelength BOTDR with BFL and passive depolarizer (AWG = arbitrary waveform generator, PC = polarization controller, PBS = polarization beam splitter, PBC = polarization beam combiner, PMF = polarization maintaining fiber, ISO = isolator, PD = photodector, DAQ = data acquisition, ES = electrical spectrum).

In 2017, Lalam et al. further analyzed the principle of WDT in BOTDR in detail and experimentally validated its enhancement effect on the SNR of BOTDR, where an enhanced SNR up to 3.92 dB was provided by three-wavelength diversity technique [49]. By combining the WDT and a low-cost passive depolarizer, the SNR was further improved by 4.85 dB compared with the conventional BOTDR system [50]. Then, they embedded a Brillouin fiber laser (BFL) into the BOTDR system as local oscillator to reduce the receiver bandwidth in the order of a few MHz. Using the proposed techniques above, they eventually obtained 5.1 dB SNRI over a 50 km sensing fiber with a spatial resolution of 5 m, which was equivalent to a 180% improvement compared with the conventional BOTDR system [51]. In 2018, Zhang et al. theoretically analyzed the channel capacity of WDM-based BOTDRs in detail by considering the fiber dispersion and nonlinear effects in sensing fibers. According to the numerical simulation, 7-wavelength and 11-wavelength BOTDR could respectively reach 7.2 dB and 8.4 dB SNRI over a 23-km-long fiber without obvious spatial degradation and nonlinear impairment [52]. The schematic block diagram of a multi-wavelength BOTDR with BFL and passive depolarizer is shown in Figure 6.

#### 2.3.2. Improvement of Probe Pulses Features

**Figure 7:**
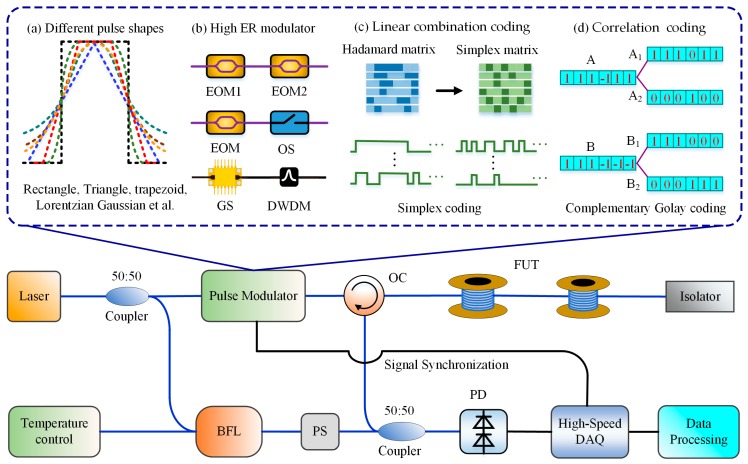
SNR enhancement of BOTDR by improving features of probe pulses: (**a**) different pulse shapes; (**b**) high ER modulator; (**c**) linear combination coding; (**d**) correlation coding. (EOM = electro-optic modulator, OS = optical switch, GS = gain switch, DWDM = dense wavelength division multiplexer, OC = optical circulator, FUT = fiber under test, BFL = Brillouin fiber laser, PS = polarization scrambler, PD = photodetector, DAQ = data acquisition).

The features of probe light pulses have a significant influence on the SNR of BFS, including the shape of a single pulse, the extinction ratio and even the coded pulse sequence, based on which a variety of schemes were proposed for BOTDR SNR enhancement, as shown in Figure 7.

It has been reported that the probe pulse shape affected the system SNR. In 2013, Hao et al. investigated theoretically and verified experimentally how the different modulated pulse shapes influenced the profile of spontaneous Brillouin scattering spectrum and the SNR of BOTDR sensing system by changing the modulation pulse format with the same pulse width. The tested pulse shapes included the rectangle, triangle, trapezoid, Lorentzian, Gaussian and hyperbolic-secant, as shown in Figure 7a. The experimental results indicated that the SNR of Brillouin power spectrum for trapezoidal pulse and triangular pulse was increased by 3 dB and 4.8 dB relative to that for the rectangular pulse with the same pulse width of 200 ns [53], and the highest measurement accuracy and the longest sensing range were provided by the Lorentz pulse [54]. This result provides a helpful guide for researchers to choose appropriate probe pulse shapes.

Moreover, the SNR of BOTDR can be improved by enhancing the extinction ratio (ER) of probe pulses, as shown in Figure 7b. In 2013, Lu et al. proved theoretically and demonstrated experimentally the positively-correlated relationship between the ER of probe pulse and the SNR of BOTDR. Then, they built a two-cascaded electro-optic modulator (EOM) to increase the probe pulse ER from 25 dB to 50 dB. The experimental results indicated that the SNR of BOTDR at a 23.9 km fiber end was increased by ~8 dB and the BFS measurement uncertainty correspondingly decreased from 6.16 MHz to 2.09 MHz [55]. In 2014, Zhang et al. further introduced a novel pulse modulation scheme combining an EOM with a synchronous optical switch (OS), which gave an improvement in the ER from 35 to 65 dB. The maximum uncertainty of measured BFS was reduced from 5.2 MHz to 0.8 MHz at the end of a 48.5 km sensing fiber with a 25 m spatial resolution [56]. Recently, Bai et al. proposed a scheme utilizing gain switch (GS) with a dense wavelength division multiplexer (DWDM) to enhance the SNR of BOTDR [57]. The root-mean-square error of the measured BFS at 9.941 km of the sensing fiber decreases from 2.49 to 0.78 MHz with the pulse ER increased by 16.21 dB. Meanwhile, the measurement distance of BOTDR utilizing the gain-switched modulation can be extended from 10.75 to 27.5 km with a 1 m spatial resolution under the same measurement conditions compared to the conventional EOM.

Besides, pulse coding has gradually become an effective technique to improve the SNR of BOTDR and attracted more and more attention, because it can increase the energy of the incident signal, meanwhile without distortion of spatial resolution. That is, the pulse coding can improve the system SNR by the coded gain. Of course, an optimum code length for a specific system should be calculated by allowing for all system noise sources [58]. Coding schemes mainly include two general groups: linear combination codes and correlation codes [59].

Simplex code belongs to linear combination codes, and the coding process was finished respectively based on Hadamard or Simplex matrix, as shown in Figure 7c. As early as in 2008, Soto et al. proposed a Simplex-coding Brillouin-based distributed temperature sensor using the Landau–Placzek ratio (LPR) scheme. The probe pulses consisted of 127-bit simplex codes and 7-dB SNRI was provided over a 21 km sensing fiber with 40 m spatial resolution [60]. To expedite the data processing of pulse coding/decoding technique, Fan et al. designed a dedicated hardware system based on field programmable gate array (FPGA) using Simplex codes and optimized the processing algorithm, which was able to complete Simplex coding/decoding with less time consumption [61]. In 2014, Hao et al. combined the Simplex pulse coding and the digital coherent detection technique in BOTDR, and a 3.5 dB SNR enhancement was obtained by 31-bit simplex pulse code comparing with the single pulse [62]. In 2010, Lu et al. firstly utilized the Hadamard sequence as probe pulses. They launched a 16-bit Hadamard code light into a 37 km optical fiber with the peak power of only −13 dBm and successfully obtain the BFS distribution over the full range, which preliminarily presented the enhancement effect of Hadamard pulses on the SNR of BOTDR [63].

Complementary Golay code belongs to correlation codes shown in Figure 7d, and the process of coding and decoding were finished based on the correlation operation between the probe light code and the backscattering signal. In 2012, Li et al. stimulated a heterodyne-detection BOTDR sensing system using 128-bit Golay complementary sequences as probe pulses, which demonstrated the SNRI arising from the coded gain [64]. In 2017, Wang et al. achieved a high-performance BOTDR sensor by combining the complementary coding for higher SNR with the fast Fourier transform (FFT) technique for higher measurement speed. The experimental results over a 10 km single-mode fiber showed that the proposed scheme achieved a spatial resolution of 2 m with a frequency uncertainty of 0.37 MHz. Theoretically, the measurement time can be more than ten times faster than traditional frequency sweeping method [65].

#### 2.3.3. Optical Link Optimization

In this section, we summarize some recent SNR enhancement schemes by optimizing the optical link, such as introducing the slow-light Mach–Zehnder interferometer (MZI) for sensitivity enhancement, MZI-based depolarizer for polarization noise suppression, the pumped amplifier for enlarging the dynamic range, the single-photon detector (SPD) for higher detection sensitivity and the self-heterodyne detection for eliminating the side effect of laser frequency instability.

**Figure 8:**
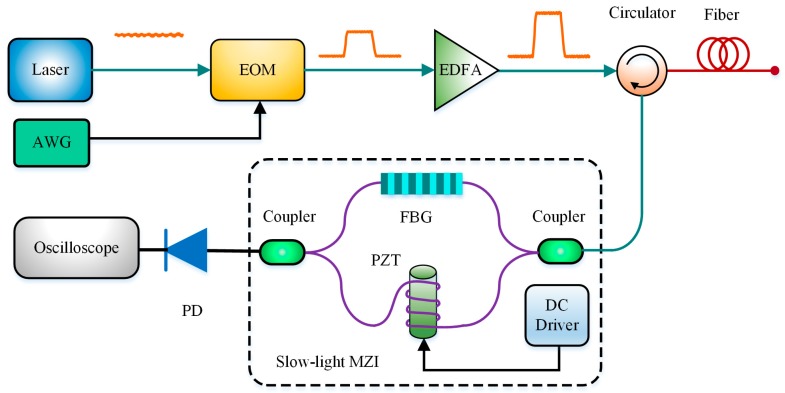
BOTDR scheme with the slow-light MZI.

As early as in 2000, the MZI was introduced in BOTDR for frequency demodulation with advantages of simple structure and low cost [29]. For further eliminating system noise, Zhao et al. in 2013 inserted a fiber grating (FG) into one of MZI arms to build a slow-light MZI. By designing the suitable structure parameters, the FG could work not only as a slow-light generator to enhance the measurement sensitivity, but also as an optical filter to reduce the power of noise light at the same time. The experimental results showed that the relative output power sensitivity and the phase sensitivity could be enhanced 20 times obviously [66]. Subsequently, they also proved that both aforementioned sensitivity could be further improved by using a fiber Bragg grating (FBG) with Gaussian index profile [67]. The BOTDR scheme with the slow-light MZI is shown in Figure 8.

**Figure 9:**
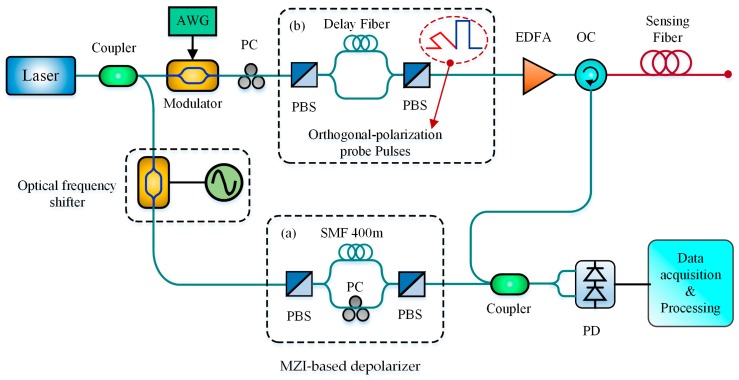
MZI-based configuration for polarization noise suppression in BOTDR.

Besides, the MZI can be also utilized to build a depolarizer to reduce polarization noise. In 2012, Wang et al. designed a passive polarization depolarizer based on the delay-MZI in BOTDR, as shown in Figure 9a. By utilizing the passive depolarizer, the polarization noise was decreased by 96% and the temperature measurement error of 1.0 °C was obtained with the spatial resolution of 3 m at the end of a 24.5 km optical fiber [68]. Similarly, Cao et al. utilized the delay-MZI with two optical polarization beam splitter (PBS) to produce probe pulses with perpendicular polarizations, as shown in Figure 9b [69]. This technique was proved to mitigate polarization fading in BOTDR and was named as orthogonal polarization probe BOTDR (OPP-BOTDR), based on which a high-accuracy BFS measurement was achieved over a 30 km optical fiber. The MZI-based configuration for polarization noise suppression in BOTDR was schematically demonstrated in Figure 9.

As is well known, both the pulse pump light and the Brillouin backscattering light suffer from transmission attenuation in long distance BOTDR, which limits the sensing distance. Hence, a pump optical amplifier was inserted during the sensing fiber to amplify both the pulse pump light and the spontaneous Brillouin back-scattering light simultaneously, for enhancing the dynamic range (DR) and extending the sensing range. In 2014, Wang et al. utilized added a pumped EDFA in testing fiber to prolong the sensing distance. It was proved that the DR and temperature resolution with backward pumped EDFA had a better performance than the forward pumped EDFA. The experimental results showed that a temperature error of 0.5 °C and a spatial resolution of 10 m were obtained with EDFA over 80 km fibers, while the efficient sensing distance was only 50 km without the pumped EDFA [70].

**Figure 10:**
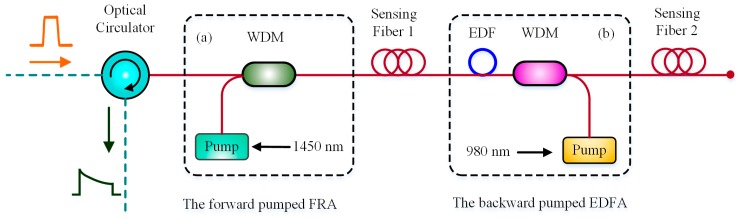
Pump optical amplifier in BOTDR: (**a**) forward pumped FRA; (**b**) backward pumped EDFA; (WDM = Wavelength division multiplexer, FRA = Fiber Raman amplifier, EDF = Erbium doped fiber).

Similarly, Song et al. added a pumped Raman amplifier before the sensing fiber. By adjusting the probe pulse power and the pump power of Raman amplifier, they achieved a 100-km sensing distance with the spatial resolution of 10 m and the temperature resolution of ±3 °C [71]. The location of backward pumped EDFA and forward pumped Raman amplifier is shown in Figure 10.

**Figure 11:**
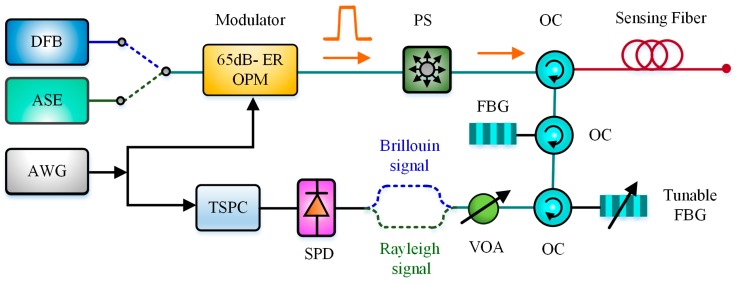
Brillouin temperature fiber sensor based on SPD and RASR (OPM = Optical pulse modulator, SPD = Single-photon detector, TSPC = Time-correlated single-photon counting, VOA = Variable optical attenuator, PS = Polarization scrambler).

Because the Brillouin backscattering light is relatively weak, the sensitivity of PD has always been a dominant factor that affects the SNR of BOTDR. With the development of detector manufacturing technology, the single-photon detector (SPD) has become commercialized. In 2016, Xia et al. utilized a SPD in BOTDR to enhance the DR due to its high sensitivity, as shown in Figure 11 [72]. The DFB source was for performing Brillouin backscattering power measurement and the ASE source was for measuring Rayleigh backscattering power. Benefiting from two cascaded FBG 1 and FBG 2, the Brillouin Anti-Stokes signals could be separated from the Rayleigh backscattering. The temperature information could be obtained by Rayleigh/Anti-Stokes ratio (RASR). The 42.5-km sensing length was achieved with a spatial resolution of 1.2 m and the temperature error of 1.7 °C.

**Figure 12:**
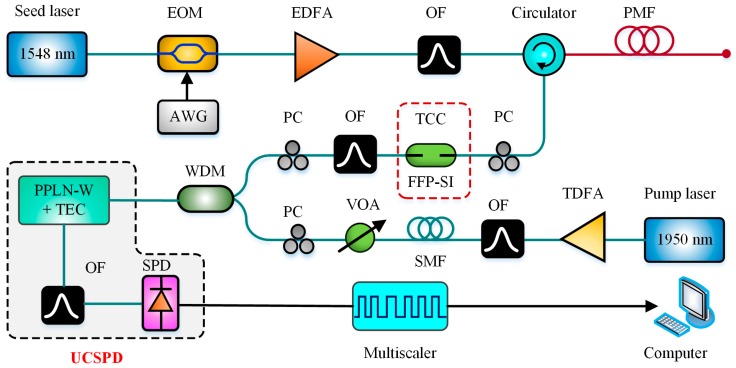
Scanning-type BOTDR based on UCSPD and FFP-SI (OF = Optical filter, PMF = polarization maintaining fiber, TDFA = Thulium doped fiber amplifier, TCC = Temperature controlled chamber, TEC = Thermoelectric cooler, FFP-SI = Fiber Fabry–Perot scanning interferometer, PPLN-W = Periodically poled lithium niobate waveguide, UCSPD = up-conversion single-photon photon detector).

However, only the power of the Brillouin backscattering was monitored as reported in [72]. For measuring both the center frequency and power change of BGS, Xia et al. utilized an up-conversion single-photon photon detector (UCSPD) and an all-fiber structure Fabry–Perot scanning interferometer (FFP-SI) to develop a scanning-type BOTDR in 2016 [73]. For higher-quantum-efficiency photon detection, the Brillouin backscattering at 1.5 μm was up converted into near-infrared wavelength at 863 nm utilizing a 1950-nm pump laser. Besides the high-resolution FFP-SI was designed for scanning BGS to seek peak BFS. Eventually, the temperature precision of 1.2 °C was obtained over 9 km polarization maintaining fiber. The scanning-type BOTDR based on UCSPD and FFP-SI is schematically presented in Figure 12.

**Figure 13:**
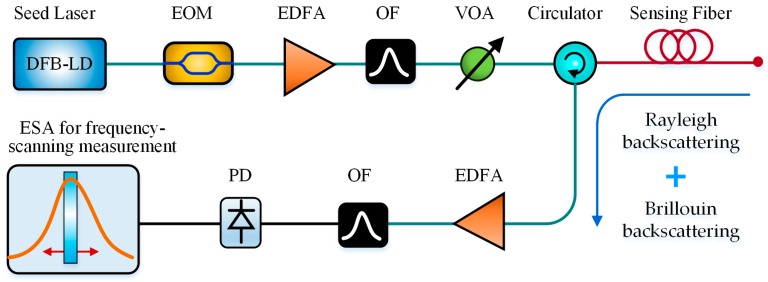
Configuration of the SH-BOTDR (VOA = Variable optical attenuator, OF = Optical filter, ESA = Electrical spectrum analyzer).

Coherent heterodyne detection is typically used in a BOTDR [17,18]. However, the frequency instability of the seed laser always degrades the measurement accuracy of BOTDR. To eliminate the side effect of laser frequency instability, Li et al. in 2017 proposed a simple BOTDR based on self-heterodyne detection between the Rayleigh and Brillouin scattering lights, without the local reference light [74]. Under the same measurement condition, the observed maximum error in temperatures for the self-heterodyne BOTDR (SH-BOTDR) was 1.2 °C, which was much better than that of 7.9 °C for the typical coherent-heterodyne BOTDR. Hence, the self-heterodyne BOTDR features not only the compact system structure but also the improved BFS measurement accuracy. Figure 13 presents the configuration of the self-heterodyne BOTDR.

#### 2.3.4. Demodulation Algorithm Optimization

In the BOTDR, the curve fitting algorithm and FFT were two commonly-used methods for obtaining BGS or BFS [17]. In recent ten years, researchers have made some new improvements to the BGS fitting algorithm and FFT, respectively, for higher BFS measurement accuracy. Meanwhile, some novel demodulation algorithms were also investigated for LPR-based BOTDR or BOTDR data post-processing.

In 2013, Zhang et al. proposed an improved Levenberg–Marquardt (LM) algorithm based on finite element analysis, named as the FEA-LM algorithm. Compared with the conventional LM algorithm, the novel one could perform better with a wider range of initial values [75]. Further, they proposed a new BGS analysis method in 2016 based on the particle swarm optimization (PSO) combining an adaptive inertia weight and chaos optimization, named as AIW-CPSO algorithm [76]. The simulation analysis and experimental results show that the fitting outcomes obtained by AIW-CPSO were matched well with the real parameters and the BFS error was reduced. The fitting degree of 0.9956 was achieved better for FEA-LM algorithm. Table 1 lists the fitting performance indicators of the two aforementioned different algorithms.

Besides, multi-peak BGS sometimes occurred during the BOTDR measurement. For instance, Liu et al. in 2014 reported a three-peak BGS due to the frequency-shift characteristics of an acoustic optical modulator (AOM) [77]. Hence, for further improving the accuracy of fitting accuracy for multi-peak BGS, Zhao et al. in 2015 investigated a novel signal-processing method to extract multiple parameters from the multi-peak BGS, including the power gain, BGS linewidth and BFS. They described the details of how to segment data, obtain the initial values for further fitting and build the objective function for the multi-peak BGS. It was demonstrated experimentally that the proposed method converged rapidly to the optimal solution and the extracted parameters were reliable with high accuracy [78].

In addition to the LM algorithm, a quadratic least-squares fitting process can be also utilized to estimate BGS. In 2013, parabolic fitting was firstly applied to BFS derivation by experimental validation [79]. Yu et al. in 2016 adopted the Monte Carlo method to numerically prove that the second order polynomial fitting method was capable of improving the BFS measurement accuracy [80]. In 2018, Zheng et al. deduced a new formula for estimating BFS measurement, allowing for both the data length of fitted points and the center deviation of the data range relative to the real frequency peak. Based on the above analysis, they proposed an iterative fitting method to improve the BFS measurement accuracy and enhance the SNR of the BOTDR system [81]. Besides, three different formulas of BFS standard deviation were respectively deduced in [79,80,81] and listed in Table 2 by considering different influence parameters.

Furthermore, the quadratic time-frequency analysis (QTFA) and auto-regressive (AR) spectral estimation technology are likewise capable of analyzing the Brillouin scattering spectrum in BOTDR, with better performance than FFT. Yao et al. in 2012 proposed a QTFA method in BOTDR based on Choi-Williams distribution (CWD) [82]. This signal processing algorithm mitigated the BFS measurement error due to the Heisenberg-Gabor uncertainty principle, hence leads to improvement of frequency accuracy without worsening spatial resolution. The experimental results showed that 3 times BFS accuracy was obtained comparing with traditional FFT or scanning-frequency method. In 2018, Huang et al. applied auto-regressive (AR) spectral estimation technology to the analysis of a Brillouin backscattering spectrum based on the Burg algorithm. The measurement accuracy was improved three times compared to that obtained by conventional FFT at a moderate spatial resolution [83].

Additionally, Pradhan et al. in 2016 presented Fourier wavelet regularized deconvolution (FourWaRD) algorithm to enhance the DR of LPR-BOTDR. They performed a numerical simulation based on the FourWaRD algorithm and LPR method for distributed strain extraction, and the 49.1 dB DR was achieved over a 70 km sensing fiber with a spatial resolution of 10 m and the launched pulse peak power of only 10 mW [84]. For high-accuracy BOTDR data post-processing, Soto et al. in 2016 proposed a two-stage adaptive algorithm to extract accurate strain profile from the acquired low-quality BOTDR data. In the adaptive algorithm, a pseudo two-dimensional convolution filter was firstly utilized to reduce noise in distance-frequency domain, then an exponential smoothing technique was utilized in time domain to eliminate temporal noise. Combining the aforementioned two-stage data processing, a SNR enhancement of 18 dB was obtained and experimentally verified by its application on real BOTDR data from an underground mine [85]. Table 3 summarizes the optimized demodulation algorithms used in BOTDR for high accuracy.

### 2.4. Measurement Speed

In most conventional BOTDRs, a frequency scanning configuration is essential to obtain the evolution of BFS by measuring the signal power at every frequency point step by step and performing BGS fitting [12]. Obviously, it needs a long time to finish one-time measurement because tens of frequency points have to be measured. However, a fast system response time is expected for some applications, such as the quick recognition of the strain/temperature changes in Brillouin sensing [86,87,88]. Hence, various schemes have been proposed to improve the measurement speed of BOTDR. In this section, we firstly listed some optimized algorithms for accelerating the signal measurement and curve fitting, then introduced FFT technique and its improvements for faster speed, finally some new-type BOTDRs without the request of frequency scanning were reviewed.

Recently, some optimized algorithms for accelerating the signal measurement and curve fitting were proposed. Ding et al. in 2010 employed pattern recognition technology to handle the mass of data of BOTDR. By analyzing both the mean and standard deviation of the characteristic vector comprehensively, the abnormal area was located fast and accurately [89]. In 2012, Wan et al. applied wavelet packet denoising technology to BGS fitting, which allowed BGS to be scanned with a larger frequency interval and without degradation of fitting accuracy. Hence, the measurement and fitting time were both reduced [90]. In 2014, Yang et al. designed a digital envelope detector (DED) based on a fast generalized harmonic wavelet transform (GHWT) algorithm to expedite the signal envelope extraction from the time-domain trace, named as GHWT-DED [91]. Comparing with the before-reported Hilbert transform [92] and Morlet wavelet transform [93], its demodulation speed can be accelerated by 39.1% and 24.9%. In 2015, Wang et al. utilized the similarity matching method (SMM) for the fast and high-accuracy recognition of the BFS change, which was obtained mainly through the correlation-coefficient-based similarity measurements [94]. The speed of data processing was 120 times faster and the BFS accuracy was 3 times better than the conventional curve fitting method. In 2018, Abbasnejad and Alizadeh proposed the moving average filter and cross-correlation denoising technique for BFS estimation, which was implemented on a FPGA-based hardware platform. This FPGA-based BFS estimator reduced the average run time by more than 2400 times compared with the conventional LM fitting method [95]. Table 4 reviews the aforementioned algorithms to improve the measurement speed of BOTDR.

As early as in 2007, the FFT technique was utilized in BOTDR for quasi-real-time temperature or strain measurement over a 12.5 km sensing fiber with 1024 averages taken in only 1 second, eliminating the time-consuming frequency scanning process [96]. In 2014, Tu et al. downconverted the BGS to the intermediate frequency region and employed short-time Fourier transform (STFT) to reconstruct BFS from the recorded time-domain data dynamically. Eventually, a 16.7 Hz strain variation over a 270 m sensing fiber was realized [97]. In 2017, Li et al. performed a dynamic strain measurement based on the STFT-BOTDR by detecting the small gain stimulated Brillouin scattering (SBS), instead of detecting spontaneous Brillouin scattering in a conventional BOTDR [98]. They stimulated small gain SBS in BOTDR through appropriately increasing the pulse power, which leaded to the power and SNR enhancement of Brillouin scattering. Hence the averaging number was reduced and the measurement speed was improved. The experimental results showed that a 60-Hz vibration at the end of a 935-m fiber is detected.

Except for FFT, other new-type BOTDRs without the request of frequency scanning were also investigated for dynamic measurement, such as MZI-based direct-detection (D-BOTDR), self-delayed heterodyne BOTDR (SDH-BOTDR), slope assisted BOTDR (SA-BOTDR) and double-edge detection BOTDR (DE-BOTDR).

**Figure 14:**
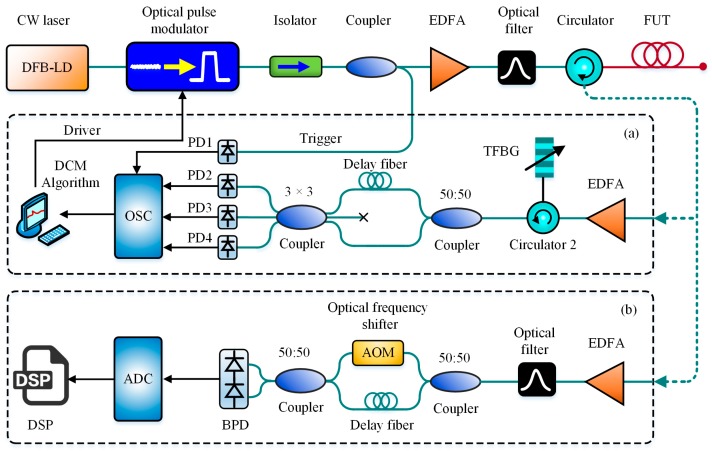
Schematic block diagram of: (**a**) MZI-based D-BOTDR; (**b**) SDH-BOTDR; (OSC = Oscilloscope, DCM = Differentiate and cross-multiply, AOM = Acousto-optical modulator, BPD = Balanced photodetector, ADC = Analog-Digital Converter, DSP = Digital signal processor).

In 2013, Masoudi et al. demonstrated a D-BOTDR by using an imbalanced Mach-Zhender interferometer (MZI) with a 3 × 3 output coupler for the use of the differentiate and cross-multiply (DCM) demodulation scheme [99]. The technique was capable of converting the BFS change to intensity variation for measurement acceleration. A strain precision of ±50 με over 2 km of sensing fiber with a SR of 1.3 m was presented with 2 Hz sampling rate. In 2015, Koizumi et al. applied the self-delayed heterodyne detection to BOTDR for high-speed strain measurement, which required no frequency sweeping and was named as SDH-BOTDR. The method was realized mainly based on a delayed interferometer composed of the delay fiber in one arm and an acoustic-optical modulator (AOM) as an optical frequency shifter in the other arm. The repetition rate reached to 25 Hz along 1 km of sensing fiber with a SR of 1.5 m [100]. The schematic block diagram of MZI-based D-BOTDR and SDH-BOTDR are presented in Figure 14a,b, respectively.

As is reported in [101,102], the slope assisted detection has been early proposed and developed in BOTDA for dynamic measurement since 2011. Similarly, Maraval et al. in 2017 transplanted this technique into a coherent BOTDR to build a SA-BOTDR, by modulating the reference frequency of local optical oscillator to be at the positive extremum slope of the BGS (named as working frequency) [103].

**Figure 15:**
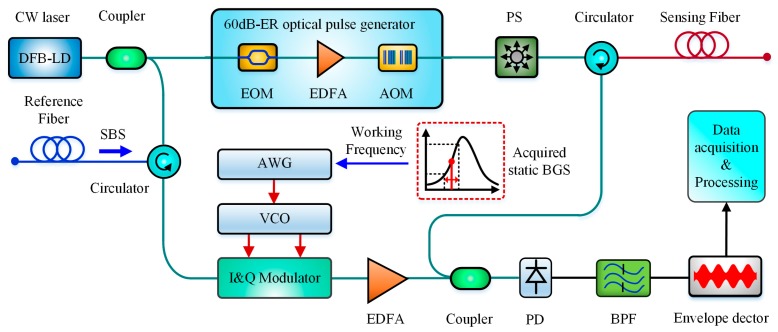
Schematic blockdiagram of SA-BOTDR (PS = Polarization scrambler, AWG = arbitrary waveform generator, VCO = Voltage control oscillator, BPF = Band-pass filter).

The working frequency was confirmed according to the acquired static BFS by one-time complete frequency-scanning method before dynamic measurement, but after it was confirmed, any change of BFS would proportionally result in a variation of the output amplitude at the working frequency, which was capable of dynamic measurement without frequency scanning any more. The acquisition rate of 7.6 Hz over 100 m optical fibers was achieved with a strain error of ±40 με and with a spatial resolution of 1 m. The schematic block diagram of SA-BOTDR is shown in Figure 15.

**Figure 16:**
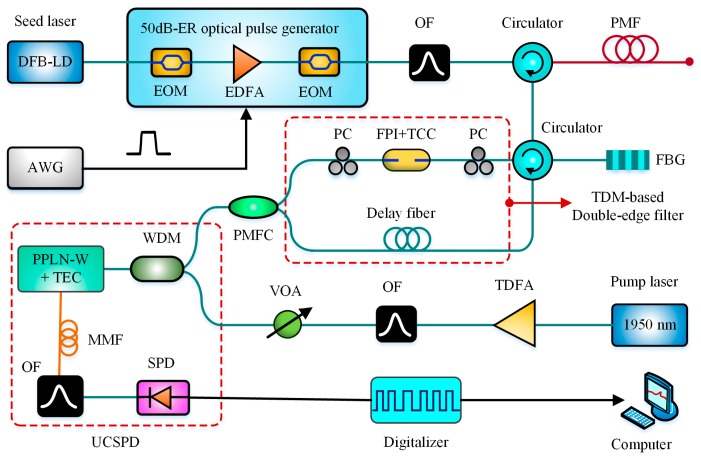
Schematic blockdiagram of DE-BOTDR (OF = Optical filter, PMF = polarization maintaining fiber, TDFA = Thulium doped fiber amplifier, TCC = Temperature controlled chamber, TEC = Thermoelectric cooler, FPI = Fabry–Perot interferometer, PPLN-W = Periodically poled lithium niobate waveguide, PMFC = polarization-maintaining fiber coupler).

The double-edge technique has been commonly utilized to measure small frequency shift Doppler lidars. In 2017, Shangguan et al. adopted this technique to design a DE-BOTDR [104]. In the scheme, a UCSPD was utilized to enhance the system SNR, and the double-edge technique transformed the strain-induced BFS change into the Brillouin signal intensity variation at two different edges of the edge filter. Besides, the time-division multiplexing (TDM) technique was performed to enable one single-channel Fabry-Perot interferometer (FPI) operating as the double-edge filter, based on its transmission curve and reflection curve. Incorporating three aforementioned techniques, a high-accuracy and fast-speed BOTDR was obtained. The experimental results demonstrated that a sampling rate of 30 Hz over a 1.5 km sensing fiber was achieved with an accuracy of ±30 με and a SR of 0.6 m. The schematic block diagram of DE-BOTDR is shown in Figure 16.

### 2.5. Cross-Sensitivity

It is well known that the BFS change is linearly sensitive to both temperature and strain variation. Hence, Brillouin distributed sensors intrinsically suffer from the temperature-strain cross-sensitivity issue [105,106], which can severely degrade their reliability in real applications. For purpose of solving this problem, many researchers have originally proposed to measure both the BFS and Brillouin intensity variations for simultaneous temperature and strain sensing [29,107]. In recent years, some hybrid sensing systems were presented and developed for addressing the cross-sensitivity problem.

**Figure 17:**
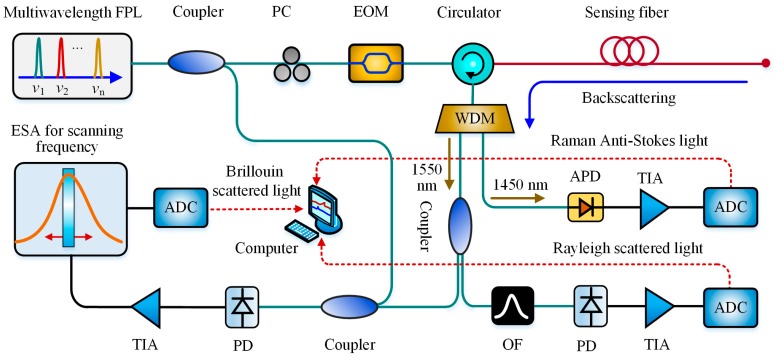
Hybrid Raman-Brillouin setup with multi-wavelength FPL (PC = Polarization controller, WDM = Wavelength diversion multiplexer, APD = Avalanche photodiode, TIA = Transimpedance amplifier, ADC = Analog-Digital Converter, ESA = Electrical spectrum analyzer).

The hybrid Raman-Brillouin-based sensor was presented as early as in 2005 for simultaneous temperature and strain measurement [108], based on which many improved scenarios were developed. Bolognini et al. in 2009 utilized a multi-wavelength Fabry–Perot laser (FPL) as the seed source in a hybrid Raman-Brillouin-based system to enhance the measurement accuracy by effectively suppressing CRN during the Rayleigh intensity measurement. The temperature distribution was derived from the power ratio of Raman anti-Stokes light to the Rayleigh-backscattering light. The strain distribution was obtained based on the linear dependence of strain variation on the BFS, where BFS distortion arising from temperature change was modified by the aforementioned Raman-based temperature distribution. Experimental results indicated that this system reached a 1.2 °C temperature resolution and the 100 με strain resolution at 25-km distance with a spatial resolution of 35 m [109]. Likewise, Xia et al. introduced the direct-detection edge technique with a twin-channel FPI into the hybrid Raman-Brillouin-based sensor for dynamical strain sensing. The simulation showed that the temperature and strain uncertainty of 0.5 °C and 20 με were obtained over within a 2 km distance, respectively [110]. Besides, the performance could be further improved by optical pulse coding techniques for SNR enhancement, such as Simplex coding [111] or cyclic pulse coding [112]. Figure 17 presents the hybrid Raman-Brillouin setup with multi-wavelength FPL.

**Figure 18:**
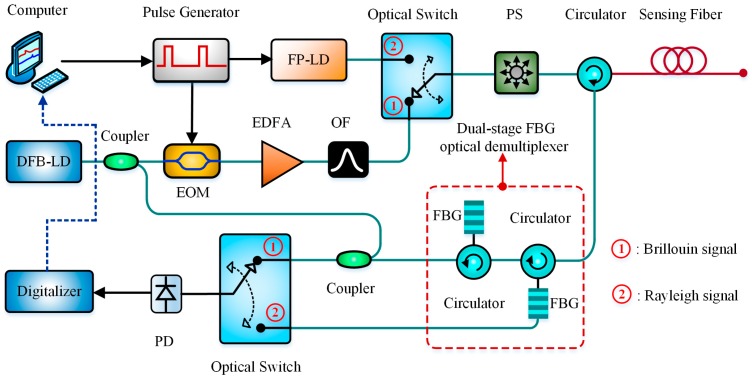
Hybrid Rayleigh-Brillouin sensor for simultaneous strain and temperature sensing with FP-LD and dual-stage FBG optical demultiplexer.

In 2013, Kim et al. designed a hybrid sensing system combining an OTDR with a BOTDR for the simultaneous measurement of strain and temperature measurement [113]. A Fabry-Perot laser diode (FP-LD) and a digital Kalman filter were introduced in the OTDR to compensate for extrinsic loss. Meanwhile, a dual-stage FBG optical demultiplexer was adopted to reduce the Rayleigh noise. Based on the aforementioned configuration, the temperature distribution was derived from the Brillouin to Rayleigh power ratio (LPR method), and the strain profile was obtained by measuring BFS change with the temperature compensation algorithm. The experimental result showed that the observed errors of temperature and strain were 0.6 °C and 50 με with a 1 m spatial resolution over a 2 km sensing distance. Figure 18 gives the schematic diagram of the hybrid Rayleigh-Brillouin sensor.

It is obvious that the aforementioned schemes are realized by utilizing the single-mode fiber. Besides, other techniques to solve the cross-sensitivity problem were proposed based on special fibers, which will be reviewed in Section 3.

### 2.6. Others

In this section, we reviewed some approaches to improve the location accuracy, simplify the system structure and reduce the electronic-bandwidth request in BOTDR. Besides, two integrated multi-function sensing systems were also demonstrated, including a hybrid BOTDR integrated with a POTDR or a phase-sensitive OTDR.

It was proved that double-peak BGS occurs over a step-shape temperature change region on the sensing fiber, which would induced location error in BFS measurement. In such cases, conventional symmetric Lorentz fitting was not suitable for restoring double peaks of BGS due to the asymmetry and deformation of the BGS. In 2015, Yu first numerically simulated and discussed experimentally this phenomenon, then applied a zero-padded STFT to BOTDR. By further applying the obtained peak power of the BGSs into a integration calculation over the effective pulse length, the position error was well compensated [114].

**Figure 19:**
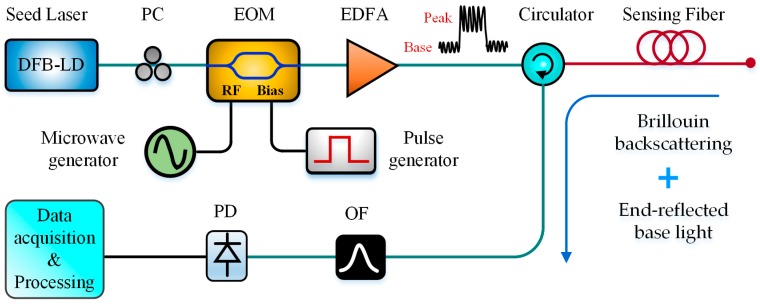
A simple single-end Brillouin-based sensors.

To simplify the system structure, some direct-detection BOTDR were proposed and developed, such as MZI-based D-BOTDR [29,66,67,99], SDH-BOTDR [100] and SH-BOTDR [74]. Similarly, Cui et al. in 2009 demonstrated a simple single-end Brillouin-based sensor, which can obtain time-domain and frequency-domain information without coherent detection. In the scheme, only one optical beam was modulated simultaneously by the pulse and microwave signals through the RF port and bias port of EOM, respectively. The pulse peak part of injected light was utilized as the pump and the modulated pulse base reflected from the far end was treated as the probe. This configuration provided a single-end Brillouin gain spectrum analysis technique, which can be also treated as a direct-detection BOTDR technique, as shown in Figure 19 [115]. Besides, Breteler et al. designed an integrated optical readout unit for BOTDR by integrating the narrow-linewidth laser, photodiodes, an optical mixer, an optical frequency tuner and a switching fabric on a 6×3 mm an optical chip. The technique allows an extremely-compact design and a sizeable cost reduction compared to present commercial BOTDR systems [116].

**Figure 20:**
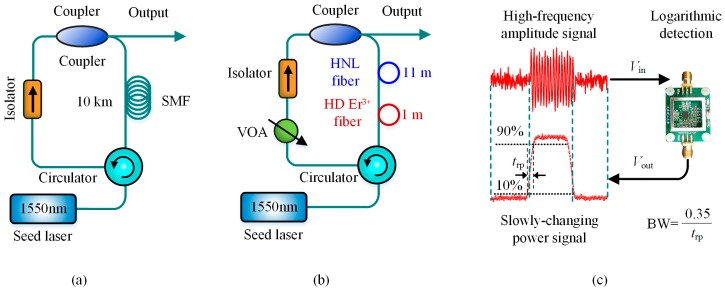
Some techniques to reduce the electronic-bandwidth request in DAQ of BOTDR: (**a**) conventional BFL; (**b**) improved BFL; (**c**) logarithmic detection scheme; (VOA = Variable optical attenuator, HNL = high nonlinear, HD = high-doped).

The BGS in single-mode fiber (SMF) has been observed as a Lorenz spectral profile with the Brillouin frequency shift (BFS) of ~11 GHz at the wavelength of 1550 nm [40]. If the BGSs are detected directly by the data acquisition (DAQ) device, the bandwidth and sampling rate have to be respectively more than 11 GHz and 22 GSa/s according to the Nyquist sampling theorem. To avoid having to use such wide-bandwidth DAQ, various schemes have been proposed in BOTDR to reduce electronic bandwidth. It was previously reported that the Brillouin fiber laser (BFL) can be treated as the local reference light in traditional heterodyne-detection BOTDR to reduce the electronic bandwidth request of DAQ from ~11 GHz to hundreds of MHz [51,96,117]. However, the traditional BFL suffered from the very large volume due to the essential reference fiber with the length of a few or tens of kilometers. Hence, Hao et al. in 2012 designed an improved BFL in a compact size, in which the too long reference fiber was replaced by the high-doped (HD) Er^3+^ fiber and high nonlinear (HNL) fiber with only 11-m length. The beat frequency was reduced to ~420 MHz based on the compact BFS [118], which performed well in subsequent BOTDR research [46,53,62]. For further lowering the electrical bandwidth request of DAQ, Bai et al. in 2018 presente a logarithmic detection scheme with short pulse response time and high measurement accuracy, which was capable of converting the high-frequency amplitude signal of Brillouin backscattering to slowly-changing power signal. Hence, the DAQ bandwidth was reduced to ~50 MHz for 1-m spatial resolution and the BFS accuracy of 0.67 MHz at 100-m end of ~ a 10 km sensing fiber was obtained [119]. Figure 20 shows the conventional BFL, improved BFL and logarithmic detection scheme to reduce the electronic-bandwidth demand in DAQ of BOTDR.

**Figure 21:**
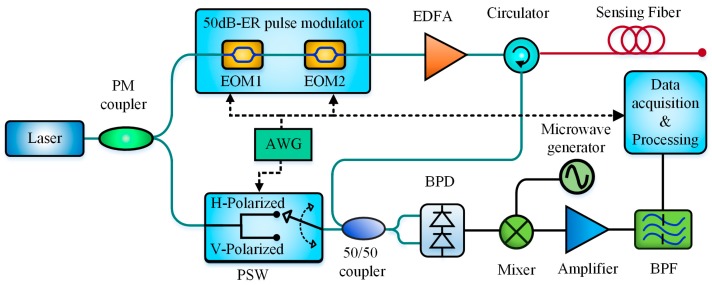
Schematic block diagram of DFSVS incorporating BOTDR and POTDR (PM = Polarization maintaining, PSW = Polarization switch, AWG = Arbitrary waveform generator, BPD = Balanced photodetector, BPF = Band-pass filter).

To measure the distributed strain and vibration at the same time, Feng Wang et al. in 2013 integrated the BOTDR with the POTDR to perform a distributed fiber strain and vibration sensor [120], as shown in Figure 21.

**Figure 22:**
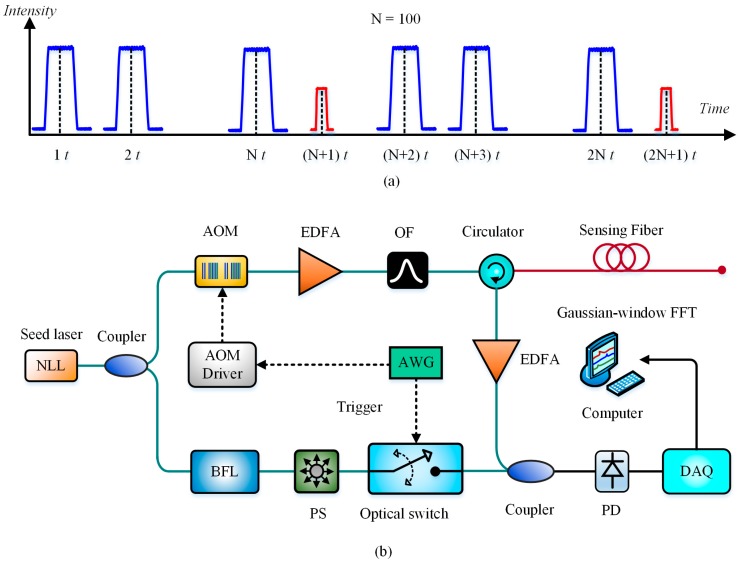
Schematic block diagram of hybrid BOTDR and phase-sensitive OTDR: (**a**) injected pulse sequence, (**b**) experimental setup (AOM = Acoustic optical modulator, OF = Optical filer, NLL = Narrow-linewidth laser, AWG = Arbitrary waveform generator, BFL = Brillouin fiber laser, PD = photodetector, DAQ = Data acquisition).

Besides, Zhang et al. in 2016 proposed a multi-parameter fiber sensing system with the hybrid BOTDR and phase-sensitive OTDR, as shown in Figure 22.

As a conclussion, the Figure-of-merit for BOTDR in the last decade is shown in Table 5.

The scheme featured the combined utilization of a frequency-scanning microwave generator and a polarization switch (PSW). The former was to perform the frequency scanning for BOTDR. The latter was to not only fix the polarization state of the reference light for POTDR detection, but also to switch the polarization state orthogonally for obtained the polarization-state-free Brillouin power distribution by summing up the two Brillouin signal acquired at X-Polarized and Y-Polarized state when the microwave generator was tuned to a certain frequency. Experimental results indicated that the BFS measurement of 0.2 MHz and the spatial resolution of 10 m was obtained over a 4 km sensing distance. For vibration detection, the frequency measurement range was up to 0.6 kHz with the frequency resolution of 2.5 Hz. In the scheme, the probe pulses were modulated in both the width and intensity. The probe pulse with peak power of 14 W and width of 30 ns was utilized for vibration detection based on the phase-sensitive OTDR, while the probe pulse with peak power of 3 W and width of 8 ns was utilized for temperature and strain measurement based on the BOTDR. The modulated probe pulses were injected into the sensing fiber in the proportion of 100:1, i.e., one hundred 30-ns pulses were firstly launched for vibration detection then followed by one 8-ns pulse for BFS acquisition, as shown in Figure 22a. The experimental setup is demonstrated in Figure 22b. Among this, an optical switch triggered by the AWG was introduced to select the Brillouin backscattering beating with the reference light from the Brillouin fiber laser. During the data processing, the Gaussian-window-based FFT was performed to ensure high spatial resolution of BOTDR. The experimental results verified that up to 4.8 kHz vibration with 3 m spatial resolution was achieved over 10 km sensing distance, as well as a high 0.8 m spatial resolution for BOTDR [121].

## 3. Novel-Fiber-Based Brillouin Sensing

As is mentioned in Section 2, the single mode silica fiber is the most commonly-used sensing fiber in BOTDR due to its advantages of low loss and effective cost. Nowadays, with the development of fiber fabrication technology, many new types of fibers come into being with increasingly better performance and lower cost, which bring in new performances for Brillouin sensing. In this section, we mainly reviewed the Brillouin scattering features of several novel fibers suitable for BOTDR or BOTDA, including plastic (or polymer) optical fibers (POFs), photonic crystal fiber (PCF), few-mode fiber (FMF), large effective-area fiber (LEAF), erbium-doped fiber (EDF) and multicore fiber (MCF).

### 3.1. Plastic (or Polymer) Optical Fibers (POFs)

In engineering application, the silica SMF suffers from disadvantages of installation fragility. It will break under the strain of only 5% and cannot be for large-strain deformation. Hence, as early as in 2004, Husdi et al. investigated the sensing properties of POF based on OTDR and indicated its potential in distributed SHM due to its enough ductility and flexibility [122]. This feature implied a practical advantage for health monitoring of buildings and civil engineering structures, which just agrees well with the application of BOTDR. Generally, the available POFs are classified into three types: perfluorinated graded-index POF (PFGI-POF), poly(methylmethacrylate) (PMMA)-based POF (PMMA-POF) and partially chlorinated graded-index POF (PCGI-POF). The Brillouin characteristics of each type of POF will be illustrated below.

#### 3.1.1. PFGI-POF

In 2010, Mizuno and Nakamura investigated the Brillouin scattering in PFGI-POF at 1550 nm wavelength (WL). The BFS, BGS bandwidth and Brillouin gain coefficient were 2.83 GHz, 105 MHz and 3.09 × 10^−11^ m/W, respectively [123]. It had a negative coefficient between the BFS and strain or temperature, which were −121.8 MHz/% (within the strain range of 1%) and −4.09 MHz/°C, respectively. The strain coefficient of −121.8 MHz/% was only −3.5 times as large as that in silica fibers which indicated the potential to achieve the strain-insensitive high-accuracy temperature sensing [124]. However, the propagation loss was ~150 dB/km at 1550 nm, which was about 750 times as much as single-mode fibers [123,124]. They further explored the influence of the core diameter on the Brillouin threshold power, which was estimated to be 53.3 W using 5 m PFGI-POFs with 62.5 μm core diameter (CD) [125].

Different from the silica SMF, a highly non-monotonic dependence of BFS occurs in PFGI-POFs under the large strain of more than 20%, which was revealed by Hayashi et al. in 2012. In the strain range of 0%–2.6%, the BFS moved toward lower with the strain increasing, while it moved toward higher in the strain range of 2.6%–10% and finally became almost constant in range of 8.1–18.3% [126]. This non-monotonic nature was potential for temperature sensing without strain sensitivity. In 2017, Minakawa further investigated the cross effect of strain and temperature on BFS in the PFGI-POF at 1550nm. The results showed that both the strain and temperature coefficient showed up the linear dependence on the other parameter in a certain changing range [127]. A brief summary of the performance of PMMA-POF is given in Table 6.

#### 3.1.2. PMMA-POF

Compared with PFGI-POFs, PMMA-POFs are more cost-effective and more widely used in communication applications [128]. Hence, the observation of Brillouin scattering in PMMA-POFs likewise attracts a lot of research attention.

In 2012, Hayashi et al. estimated the BFS change under different temperatures or strains based on the ultrasonic pulse-echo technique. The experimental results showed that BFS was ~13 GHz and the temperature coefficient was −17 MHz/°C at 650 nm, −14.5 times larger than that of a silica fiber at 1550 nm [128,129]. However, there was no linear relationship between the BFS and strain. Hence the PMMA-POFs had a potential to achieve the temperature detection with high precision but could ignore the cross sensitivity which emerged in the silica-based single-mode fibers (SMFs) [128]. In 2016, Minakawa et al. further studied the relationship between the BFS of the PMMA-POF and the environmental humidity. It was observed that the BFS showed a decreasing trend with the incensement of environmental humidity and temperature, which showed the potential of detecting environmental humidity using the PMMA-POF [130]. The performance of PMMA-POFs is summarized in Table 7.

#### 3.1.3. PCGI-POF

Compared with the two aforementioned type of POFs, the PCGI-POF features high thermal stability up to ~100 °C, which enables the wide-range temperature sensing based on Brillouin sensors [131]. Hence, PCGI-POFs recently attracted considerable research attention.

In 2013 Minakawa et al. firstly investigated the Brillouin characteristic of PCGI-POF at a wavelength of 1550 nm [131]. The BFS was ~4.43 GHz. The linear temperature coefficient was −6.9 MHz/°C, nearly 5.8 times as large as that of silica SMFs. This high temperature sensitivity indicated that the PCGI-POF can be used to achieve temperature detection with high precision. However, the fracture strain of this fiber was about 3%, not suitable for large-strain sensing. Further, they developed a single-end strain/temperature sensor based on multimodal interference in PCGI-POF by using Fresnel reflection from the fiber end. The experimental results verified that strain and temperature sensitivity of −122.2 pm/με and 10.1 nm/°C were obtained, respectively [132]. A brief summary of PCGI-POF performance is shown in Table 8.

### 3.2. Photonic Crystal Fiber (PCF)

Photonic-crystal fibers (PCF) can be considered as a subgroup of micro-structured optical fibers, the core cross-section of which has a complex refractive index profile originating from the distribution of air holes with different arranging pattern. Hence, the PCF provides various novel optical characteristics no existing in conventional optical fibers. Here, we reviewed multi-peak features of Brillouin gain (loss) spectrum in some PCFs, which are potential to distinguish temperature and strain simultaneously.

In 2003, for the first time, Zou and Bao et al. observed the five-peak Brillouin loss spectrum in PCF with a partially Ge-doped core [133]. The peaks were caused by different guided longitudinal acoustic modes in the PCF acting as both an acoustic waveguide and antiwaveguide. They further investigated different dependences of strain and temperature on the frequency shift of the first and third peak (Peak a and Peak c) [134]. Based on the different temperature and strain coefficients of the two peak frequencies, they realized a highly precise simultaneous distributed strain and temperature sensor with a SR of 15 cm.

In 2012, Zou et al. investigated the Brillouin scattering property of a highly nonlinear photonic crystal fiber (HNL-PCF) [135]. In this fiber, the core was Ge-doped core and triangularly-arranged by the F-doped buffer. Similarly, a five-peak Brillouin scattering spectrum was observed.

In 2017, Zhang et al. realized discrimination of simultaneous temperature and strain change by extracting the birefringence frequency shift (BireFS) of Brillouin dynamic gating (BDG) and BFS of BGS in polarization maintaining PCF(PM-PCF) [136]. Unlike the common silica fiber, temperature dependence of the BireFS in PCF was not a linear relationship but varied versus different temperature regions. The Brillouin features of the several aforementioned PCF are listed in Table 9.

### 3.3. Few-Mode Fiber (FMF)

As is well known, conventional SMF-based Brillouin sensors suffer from cross-talk between temperature and strain, because the BFS of the fiber varies with either the environmental temperature or the strain changes. Thus, the cross-sensitivity of temperature and strain has always been an imperative issue which needs to be solved in BOTDR [105]. Recently, the few-mode fiber is potentially expected to reduce the cross-sensitivity. There are several spatial modes in FMF and each mode possesses a different effective refractive index, chromatic dispersion and loss. Hence, the Brillouin scattering for various LP modes will create different BGSs and the corresponding BFSs [137,138], which always lead to different temperature and strain coefficient and hence benefit the distinction of temperature and strain.

In 2014, Li et al. utilized the programmable spatial light modulator to build a reconfigurable BOTDR and realized the measurement of distributed mode coupling in a five-mode FMF [139]. Then, they further investigated the temperature and strain coefficients of LP_01_ and LP_11_ modes, which were 1.01690 MHz/°C, 0.05924 MHz/με for LP_01_ mode and 0.99099 MHz/°C, 0.04872 MHz/με for LP_11_ mode, respectively. They proposed a FMF-based BOTDA to measure the temperature and strain simultaneously with the accuracy of 1.2 °C and 21.9 με [140].

In 2015, Weng et al. designed a Ge-doped step-index FMF and measured the temperature and strain simultaneously based on a coherent-detection BOTDR [141]. The temperature and strain coefficient was 1.29 MHz/°C and 58.5 kHz/με for LP_01_ mode, and 1.25 MHz/°C and 57.6 kHz/με for LP_11_ mode. Additionally, a Ge/F co-doped double-index HNL-FWM was proposed for further enhancing the discriminative sensing performance.

In 2017, Zhou et al. proposed a graded-index few-mode fiber (GI-FMF) with the radius of 5 μm and the maximum GeO_2_-doping concentration of 10%, which supported the two guided modes of LP_01_ and LP_11_. Due to the interaction between multiple acoustic modes, multi-peak BGS was obtained with different temperature and strain coefficient. Based on simulation results, the system accuracies for simultaneous temperature and strain measurement were 1.8 °C and 41 με, respectively [142]. Besides, the special feature Brillouin scattering in FMF was also competently for the distributed curvature sensing [143]. The Brillouin features of the several aforementioned FMFs are given in Table 10.

### 3.4. Other Special Fibers

In 2012, Liu and Bao utilized large effective-area fiber (LEAF) as the sensing fiber in BOTDA. Allowing for both the Brillouin peak and linewidth in LEAF, simultaneous temperature and strain measurement was realized [144]. Further, Lu et al. applied the LEAF to a homodyne BOTDR. They observed multiple peaks occurring in Brillouin beat spectrum (BBS) due to interaction between different modes of LEAF. By measuring the 1st and 2nd peak intensity of BBS, the temperature and strain were discriminated attributed to different strain and temperature dependences of the two beat peak power [145].

In 2013, Ding et al. investigated BGS in erbium-doped fiber (EDF) and explored the temperature and strain dependence of BFS at 1.55 μm. The experimental results verified that the BFS and its temperature and strain coefficients in the EDF with 0.72 wt ppt erbium concentration were 11.42 GHz, 0.87 MHz/K, and 479 MHz/% [146]. In 2014, they further demonstrated that the fluorescence intensity ratio (FIR), which is the ratio of the fluorescence intensities at two different wavelengths 1530 nm and 1565 nm in the experiment, was linearly dependent on temperature (with a coefficient of 5.6 × 10^−4^/°C) but almost independent of strain. Combining BFS and FIR, the discriminative strain and temperature measurement was expected to be achieved [147].

In 2016, Zhao et al. employed the multi-core fiber (MCF) to build a spatial-division multiplexed hybrid Raman and Brillouin OTDR. In the scheme, the probe pulse from a single laser was injected into two separate fiber cores through a MCF fan-in coupler for Raman and BOTDR pump, respectively. The temperature was obtained based on the spontaneous Raman scattering and the strain was acquired from BFS, which ensured the capability of discriminative temperature and strain. The measurement resolutions were estimated to be about 2.2 °C and 40 με respectively at 6 km sensing range with 3 m spatial resolution [148]. In 2018, Zaghloul et al. utilized both cores of MCF as Brillouin probe channels for BFS acquisition, and the temperature and strain were distinguished due to the different temperature and strain coefficient of BFS in two cores [149].

Moreover, the measurement accuracy of BOTDR is related closely to the features of sensing fibers. In 2018, Luo et al. tested the highly nonlinear fiber (HNLF) in STFT-BOTDR [150]. The frequency uncertainty of 0.43 MHz was achieved, which would increase to about 1.02 MHz for the standard single mode fiber under the same condition. The experimental results proved that the HNLF enhanced the SNR of BOTDR. The Brillouin features of the aforementioned EDF, LEAF and MCF are shown in Table 11.

## 4. Applications

Benefiting from the special characteristics of optical fiber, the optical fiber sensing possesses inherent engineering-applicable advantages including long sensing distance, mass measurement points, anti-electromagnetic interference, corrosion resistance and intrinsic safety. With the development of optical fiber sensing, BOTDR has gradually been an innovative technique that allows measurement of distributed strain and temperature profiles for distances of up to tens of kilometers, attributed to its particular superiority of single-end access and easy implementation. In recent decade, BOTDR was widely utilized in SHM, geological disaster prewarning and other strain/temperature-related sensing applications, such as status monitoring of transmission lines, soil temperature and ice structure, etc.

### 4.1. SHM of Large-Range Infrastructure

#### 4.1.1. Tunnel, Bridge and Pavement Monitoring

BOTDR-based strain measurement can be utilized for tunnel monitoring by detecting embedded cavities, smuggling excavation, lining deformation wall displacements, etc. For instance, Lanticq et al. utilized a soil-embedded optical fiber cable as sensing fiber of BOTDR to detect embedded cavity of the Ebersville railway tunnel in French for sinkhole erosion and soil collapse warning [151]. Klar et al. laid two fiber horizontally and vertically to build a BOTDR-based smart underground fence for prohibiting excavation of smuggling tunnels [152]. Mohamad et al. researched the twin-tunnel interaction by recorded circumferential strains of the outer tunnel utilizing BOTDR, when the parallel inter tunnel was excavating in Singapore MRT circle line 3 [153]. Webb et al. performed the state monitoring of a prestressed concrete beam-and-slab bridge located in Cambridgeshire in the UK [154]. He et al. proposed a smart bridge optical cable for bridge damage detection by combining FBG and BOTDR/A technique [155]. Liu et al. designed a health monitoring sensor for multi-layered pavement by incorporating the FBG and BOTDR sensing techniques. This configuration ensured the large-scale damage monitoring and local high accurate strain measurement [156].

#### 4.1.2. Long-Range Pipeline Monitoring

Gas or oil leakage of pipelines will always cause a temperature change of its ambient environment, and abnormal bending of pipelines will distort the surface strain distribution. Hence, BOTDR is suited to perform long-range pipeline monitoring due to its ability of temperature and strain measurement. For instance, Yan et al. proposed an enhanced BOTDR for higher-SNR oil and gas pipeline monitoring by simplex coding [157]. Mirzaei et al. demonstrated a leakage detection system for buried oil pipelines based on a BOTDR temperature sensor [158]. Similarly, Madabhushi et al. laid out temperature-sensing cables and employed BOTDR to detect heated oil leakage of subsea pipelines [159]. Feng et al. developed a BOTDR-based methodology for detecting lateral buckling of subsea pipelines [160]. Bai et al. designed a coal-bed methane pipeline safety monitoring system based on the multi-parameter optical fiber sensing technology with combination of BOTDR, ROTDR and ϕ-OTDR, among which the BOTDR was utilized to perform strain detection for deformation precaution of beyond 10 km of buried pipeline [161].

#### 4.1.3. Precast Pile Monitoring

Precast piles are widely utilized to reinforce soft soil in nearly every infrastructure engineering project, and their structural health is vital for construction safety. However, conventional strain gauges and the attached electric wires may not survive because of the harsh surroundings during the construction process. Hence, BOTDR-based sensors have been gradually utilized for precast pile monitoring due to its superiority of long distance, distributed sensing, corrosion resistance and easy implementation. For example, Lu et al. applied BOTDR to precast pile monitoring and calculated deformation and stress based on the axial and bending strain data [162]. Sun et al. attached sensing cable to the pile in a coil winding setup using polyurethane adhesives, and the expansion deformation of the pile was measured based on BOTDR strain detection [163]. Feng et al. combined the BOTDR and FBG techniques to monitor pile-shaft strain changes of two super-long steel pipe piles in offshore areas [164].

#### 4.1.4. Mine Safety Monitoring

The movement, collapse and displacement of mine roofs easily causes mine accidents. Deformed strain distribution of mine roofs could be recognized by BOTDR for preventing the occurrence of roof collapses. For instance, Nan et al. utilized BOTDR to measure the stope roof movement and ground surface subsidence in an iron mine during the process of open stopping and subsequent backfilling mining [165]. By integrating various wiring method of sensing optical cables for different roof structures, Wang and Luan realized detection of mine roof collapse based on BOTDR strain sensing [166]. Moffat et al. employed BOTDR to monitor rock mass displacement arising from mining activities, based on a custom PVC tube with four optical sensing fibers glued on the surface rotated 90 degrees one from each other [167].

### 4.2. Geological Disaster Prewarning

BOTDR can be utilized for soil-slope stability monitoring, landslide early warning, land subsidence and ground displacements measurement. For example, Wang et al. applied the BOTDR technique to measure the strain distribution and deformation of a laboratory soil-slope model under various loads. They proved that the BOTDR was available to estimate the stability of artificial soil slopes [168]. Yin et al. exploring the feasibility of BOTDR for landslide deformation monitoring at the relocated Wushan Town, near the the Three Gorges Reservoir in China, which indicated that the average strain of the sensing fiber increased on the upper landslide [169]. Gu et al. investigated the land subsidence by combining microstructure analysis of soils and distributed optical fiber sensing. In the scheme, BOTDR, FBG-based osmometer and FBG-based extensometer were merged for both full-distributed and quasi-distributed monitoring [170]. Klar et al. utilized BOTDR to measure the tunneling-induced ground displacements by monitoring a horizontally laid optical fiber at a shallow depth above a tunnel line [171].

### 4.3. Other Applications

**Figure 23:**
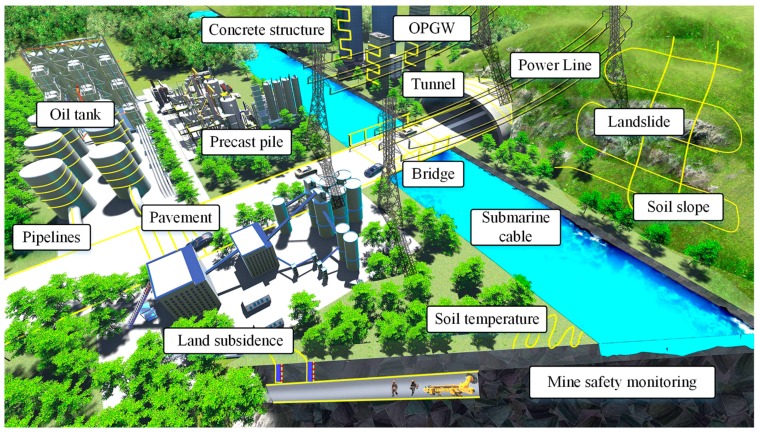
Several typical applications of BOTDR.

Luo et al. incorporated the BOTDR and FBG technologies to implement an on-line temperature and strain fiber sensing system of overhead transmission lines, in which the BOTDR system was used to measure the temperature of overhead lines and the FBG system was adopted to measure the tension of overhead lines [172]. BOTDR-based temperature and strain sensing technology provided an effective method of distortion monitoring, dynamic ampacity analyzing and icing forecasting for transmission lines, such as optical power ground wire (OPGW) [172,173,174]. Likewise, the BOTDR can be also utilized to perform on-line temperature monitoring of high-voltage submarine cable [175], and three-phase power transmission lines [176]. Besides, Zhou et al. utilized BOTDR and FBG sensors to monitor ice structure for investigating strain state and damage threshold of ice structures under uniaxial loads [177]. Lei Gao et al. designed a high-spatial-resolution BOTDR for distributed soil temperature measurement [178]. Figure 23 illustrates several typical applications of BOTDR.

## 5. Conclusions and Prospects

In this paper, we have attempted to review the new advances of BOTDR in the last decade. In terms of BOTDR performances, various technique aiming at improving the spatial resolution, SNR, measurement accuracy, measurement speed and other parameters are interrogated and discussed. Besides, the new Brillouin sensing features of novel-type fibers were summarized, including PFGI-POF, PMMA-POF, PCGI-POF, PCF, FMF, LEAF, EDF and MCF. The advanced achievements in BOTDR make it widely utilized in field engineering application. We listed its application in SHM of tunnels, bridges, pavements, pipelines, precast piles and mine roofs. It has also been utilized for soil-slope stability monitoring, landslide early warning, land subsidence and ground displacements measurement, etc. Although researchers have made huge contributions to the development of BOTDR, there are still many obstacles to be further investigated. Here, we have attempted to list three aspects needed to be focused in future research, (a)~(b) for further performance enhancement and (c) for application popularization, drawn as follows:

(a) Synchronized multi-performance improvement

It has been proved that the performance parameters of BOTDR are restricted by each other instead of being independent. For example, there is a trade-off between the spatial resolution (time-domain accuracy) and BFS measurement accuracy (frequency-domain accuracy) [31,179]. Meanwhile, the measurement speed is related to the SNR, measurement distance and measurement accuracy [100]. Hence, new theories and methods should be proposed for improving comprehensive performances of BOTDR synchronously. Recently, several artificial intelligence algorithms have been utilized in BOTDA for fast-speed and high-accuracy temperature and strain extraction without cross-sensitivity [180], such as artificial neural network (ANN) [181,182], support vector machine (SVM) [183,184,185], deep neural network (DNN) [186], which can provide new ideas for BOTDR multi-performance synchronous enhancement.

(b) Multi-function integration

Multi-function-integration optical fiber sensing interrogators have been increasingly proposed, such as BOTDR with ROTDR [108], BOTDR with POTDR [120], BOTDR with ϕ-OTDR [121], BOTDA with ϕ-OTDR [187], BOTDA with COTDR [188]. Hence, more optical fiber sensing techniques are expected to be further integrated with BOTDR for multi-parameter detection only utilizing a single fiber as sensing medium.

(c) Standardization of BOTDR application

BOTDR is capable of detecting temperature or strain, and has been widely utilized in field applications, especially for disaster prewarning. However, there are no generally-agreed or well-developed standards for BOTDR applications, which has led to the embarrassing fact that the BOTDR would not be accepted in some intelligent structures design and construction, especially for safety-relevant functions. The details of BOTDR application request to be guided and constrained by relevant industry standards, such as how to perform the field installation of sensing fiber and how to interpret the meaning of observed BFS change, etc. Hence, the proposal and development of a standard or guideline is necessary for facilitating application popularization of BOTDR.

## Figures and Tables

**Table 1:** Fitting performance indicators of the two different algorithms.

Algorithm	Highlights Technique	BFS Error/MHz	Computational Effort/s	Fitting Degree	Features
FEA-LM	Finite element analysis	0.9135	0.5274	0.9402	Robust for initial values
AIW-CPSO	PSO + Chaos optimization	0.5602	0.6665	0.9956	Better fitting degree Higher accuracy

**Table 2:** Formulas of BFS standard deviation obtained by the three fitting algorithms.

BFS Standard Deviation: *σ*	Parameters	Reference
$\sigma =\frac{1}{SNR_{A}}\sqrt{\frac{3}{4}f_{\mathrm{step}}\cdot \Delta \nu_{B}}$	*f*_step_: frequency step of data points *SNR*_A_: signal-to-noise ratio in amplitude Δ*v*_B_: the FWHM of BGS	[79]
$\sigma =0.67\times \frac{1}{SNR_{A}}\sqrt{\frac{f_{\mathrm{step}}\cdot \nu_{B}}{Q}}$	$Q=\frac{\nu_{B}}{\Delta \nu_{B}}$: *Q*-factor of BGS	[80]
$\sigma =\frac{8\sqrt{\rho^{3}q\Delta \nu_{B}f_{\mathrm{step}}}}{3\cdot SNR_{A}}$	$\rho =\frac{x_{N}}{\Delta \nu_{B}}$, $x_{N}$: frequency range of data points 3*q*/*ρ* ~ 2	[81]

**Table 3:** Optimized demodulation algorithms in BOTDR for high accuracy.

Functionality	Algorithm	Research Group	Year	Reference
Improved LM	FEA-LM	Yanshan University, China	2013	[75]
	AIW-CPSO	Yanshan University, China	2016	[76]
	Multi-peak fitting	North China Electric Power University, China	2015	[78]
Quadratic least-squares fitting	Parabolic fitting	EPFL Swiss Federal Institute of Technology, Switzerland	2013	[79]
	Second order polynomial fitting	University of Cambridge, UK	2016	[80]
Quadratic least-squares fitting	Iterative fitting	Shanghai Institute of Optics and Fine Mechanics, Chinese Academy of Sciences	2018	[81]
QTFA	CWD	Nanjing University, China	2012	[82]
AR spectral estimation	Burg	Jinan University, China	2018	[83]
LPR-BOTDR	FourWaRD	Indian Institute of Technology Bhubaneswar, India	2016	[84]
BOTDR data post-processing	Online two-stage adaptive algorithm	Universidad de Chile, Chile	2016	[85]

**Table 4:** New algorithms to improve the measurement speed of BOTDR.

Functionality	Algorithm	Research Group	Year	Reference
Mass data handling	Pattern recognition	Nanjing University of Science and Technology, China	2010	[89]
BGS fitting	Wavelet packet denoising	Nanchang Hangkong University, China	2012	[90]
Digital envelope detector	GHWT	Beihang University, China	2014	[91]
Seeking BFS change	SMM	Nanjing University, China	2015	[94]
FPGA-Based BFS estimator	Moving average filter Cross-correlation denoising	University of Tehran, Iran	2018	[95]

**Table 5:** Figure-of-merit for BOTDR in the last decade.

Research Group ^1^	Sensing Range (km)	Spatial Resolution (m)	Measurement Accuracy	SNRI or DR Yes/No (dB)	Measurement Speed Yes/No (s/Hz)	Highlights	Year	Reference
Beihang University, China	0.27	1	4 MHz	N	N	Self-heterodyne detection	2009	[115]
	2	0.1	0.5 °C, 20 με	N	N	ROTDR + BOTDR	2009	[110] ^3^
	5.1	15	N	5.5	15.2 s	GHWT-DED	2014	[91] ^2^
Cementys, Universit’e Paris Saclay, France	0.1	1	±40 με	N	7.6 Hz	Slope-assisted BOTDR	2017	[103]
China University of Geosciences, China	3	0.2	N	N	N	Differential pulse technique	2018	[40]
Chongqing University, China	10	0.8	N	N	N	BOTDR + ϕ-OTDR	2016	[121]
Indian Institute of Technology Bhubaneswar, India	70	10	125 με	49.1	N	FourWaRD algorithm	2016	[84] ^3^
Ibaraki University, Japan	0.016	0.2	±1 MHz	N	N	DP-BOTDR	2007	[34,35]
Jinan University, China	2	25	0.5 MHz	N	N	AR Burg algorithm	2018	[83] ^4^
Kyungpook National University, Korea	2	1	0.6 °C, 50 με	4	N	FPL-based OTDR + BOTDR	2013	[113]
Nanjing University, China	37	N	N	N	N	Hadamard coding	2010	[63]
	24	3	1 MHz	N	N	MZI-based depolarizer	2012	[68]
	0.36	1.6	±1 MHz	N	N	CWD algorithm	2012	[82]
	23.6	10	0.8 MHz	4.2	N	WDT	2012	[48]
	1	0.1	1.1 MHz	N	N	Iterative subdivision	2013	[42]
	50	1.5	1.8 MHz	N	N	Iterative subdivision	2013	[42]
	23.9	5	2.1 MHz	N	N	High-ER modulator	2013	[55]
	4	10	0.2 MHz	N	N	BOTDR + POTDR	2013	[120]
	48.5	25	0.8 MHz	N	N	High-ER modulator	2014	[56]
	0.27	4	45 με	N	16.7 Hz	STFT	2014	[97]
	36	10	0.92 MHz	N	N	SMM	2015	[94] ^5^
	42.5	1.2	1.7 °C	8.5	N	SPD + RASR	2016	[72]
	10	2	0.37 MHz	N	6 s	Golay coding and FFT	2017	[65]
Nanjing University of Aeronautics and Astronautics, China	23	5	0.2 MHz	8.4	N	WDT	2018	[52]
North China Electric Power University, China	2	N	N	8	N	Complementary coding	2012	[64]
North China Electric Power University, China Northumbria University, UK	9.5	13	1.2 °C	N	N	Self-heterodyne detection	2017	[74]
	10	10	1.52 MHz	3.92	N	WDT	2017	[49]
Northumbria University, UK NP Photonics, USA	25	5	0.18 MHz	4.85	N	WDT + MZI-based depolarizer	2018	[50]
	50	5	0.52 MHz	5.1	N	WDT + MZI-based depolarizer + BFL	2018	[51]
	12.5	20	N	N	1 s	FFT + BFL	2007	[96]
NTT Corporation, Japan	3	100	0.857 MHz	N	N	Low bandwidth	2009	[117]
OKI Electric Industry, Japan	1	1.5	N	N	25 Hz	SDH-BOTDR	2015	[100]
Osaka University, Japan	0.04	0.1	N	N	N	S-BOTDR,	2014	[36]
Scuola Superiore Sant’Anna, Italy	21	40	3.1 °C	7.1	N	Simplex coding	2008	[60]
Scuola Superiore Sant’Anna, Italy Shanghai Institute of Optics and Fine Mechanics, Chinese Academy of Sciences, China	30	42	5 °C	7.1	N	Simplex coding	2008	[59]
	25	35	1.2 °C, 100 με	N	N	FPL + ROTDR + BOTDR	2009	[109]
	53	32	8.8 °C, 220 με	7.4	N	BOTDR + Simplex coding	2009	[111]
	20	10	±2 MHz	N	N	BFL	2012	[118]
Shanghai Institute of Optics and Fine Mechanics, Chinese Academy of Sciences, China	5	8	N	5	N	Modulated pulse format	2013	[54]
	9.5	20	N	4.8	N	Modulated pulse format	2013	[53]
	10	10	1.5 MHz	N	N	Narrow Laser Linewidth	2013	[46]
	1	4	9.706 MHz	3.5	N	Simplex coding	2014	[62]
	30	10	N	N	N	MZI-based depolarizer	2014	[69]
Shibaura Institute of Technology, Japan	0.38	0.2	N	N	N	PSP-BOTDR	2016	[37]
	0.354	0.2	1.08 MHz	N	N	PSP-BOTDR	2017	[33]
	0.35	0.2	3.2 MHz	N	N	DCS-BOTDR	2018	[38]
South China University of Technology, China	7.8	0.4	4.1 MHz	N	N	Differential pulse technique	2016	[39]
Taiyuan University of Technology, China	10.2	5	0.595 MHz	4.35	N	Side-band detection	2016	[77]
	10	1	0.67 MHz	N	N	Logarithmic detection	2018	[119]
	10.2	1	0.805 MHz	N	N	Optimized Laser Linewidth	2019	[47]
	10	1	0.78 MHz	N	N	High-ER modulator	2019	[57]
Cambridge University, UK	1.5	3.4	N	N	N	Zero-padded STFT	2015	[114]
	0.935	4	N	N	60 Hz	Small-gain SBS	2017	[98]
University of Science and Technology of China, China	9	2	1.2 °C	N	15 s	UCSPD	2016	[73]
	1.5	0.6	± 30 με	N	30 Hz	UCSPD + DE-BOTDR	2017	[104]
University of Southampton, UK	2	1.3	±50 με	N	2 Hz	MZI-based D-BOTDR, DCM demodulation	2013	[99]
Yancheng Institute of Technology, China	80	10	0.5 °C	N	NA	Backward pumped EDFA	2014	[70]
Yanshan University, China	N	0.1	N	N	N	Pulse subdivision superposition	2017	[43]
Zhejiang University	100	40	±3 °C	N	N	Unidirectionally pumped Raman amplifier	2016	[71]

^1^ The research groups were sorted and listed alphabetically; ^2^ It is the recorded demodulation time for per envelope of the GHWT-DED with average times of 2^10^; ^3^ The parameters were obtained based on numerical simulation; ^4^ The measurement accuracy was an estimated value based on experiment results in [83]; ^5^ The sensing range of 36 km was simulated by adding attenuators with total insert loss of 6.5 dB.

**Table 6:** Brief performance summary of PFGI-POF.

Fiber Parameters	Values	Potential Application
BFS [123,124]	~2.83 GHz (WL = 1550 nm)	1. Potential use for strain-insensitive high-accuracy temperature sensing 2. For large-strain sensing
Temperature coefficient [124]	−4.09 MHz/°C	
Strain coefficient [124]	−121.8 MHz	
Brillouin linewidth [124]	105 MHz	
Threshold power [123,125]	24 W (CD = 120 μm, L = 100 m) 53.3 W (CD = 62.5 μm, L = 5 m)	
Numerical aperture [124]	0.185	
Refractive index [124]	~1.35	
Transmission Loss [127]	~250 dB/km (WL = 1550 nm)	
Strain-coefficient dependence on temperature [127]	1.5 MHz/(%·°C) (strain: 0–1.2%)	
	−0.3 MHz/(%·°C) (strain: 4.0–9.0%)	
Temperature-coefficient dependence on strain [127]	1.5 MHz/(°C %) (strain: 0–1.2%)	
	−0.3 MHz/(°C·%) (strain: 4.0–9.0%)	
	Independent (strain: >13%)	

**Table 7:** Brief performance summary of PMMA-POF.

Characteristics [128,129]	Parameters [128,129]	Potential Application
Core diameters	980 μm	1. Solving cross-sensitivity of BOTDR [128]. 2. Humidity detection [130]
Numerical aperture	0.5	
Refractive index	~1.49	
Transmission Loss	~150 dB/km (WL = 650 nm)	
BFS	~13 GHz (WL = 650 nm)	
Temperature coefficients	−17 MHz/°C (WL = 650 nm)	
Strain coefficients	No linear relationship with strain	

**Table 8:** Brief performance summary of PCGI-POF [131].

Fiber Parameters	Values	Potential Application
Core diameter	120/750 μm	Potential use for high-precision temperature sensing
Core refractive index	1.52	
BFS	~4.43 GHz (Wavelength = 1550 nm) ~10.57 GHz (Wavelength = 650 nm)	
Temperature coefficients	−6.9 MHz/°C (Wavelength = 1550 nm)	
Fracture strain	3%	

**Table 9:** Brillouin scattering features of several PCFs.

Cross Section	Fiber Parameters	Values			
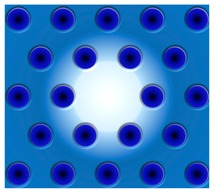 Ge-doped [133,134]	Core diameter	2.3 μm with 0.8 μm Ge-doped center region			
	Effective area	5 μm^2^			
	BFS (at 1320 nm)	Peak a: 12.054 GHz		Peak c: 13.046 GHz	
	Temperature coefficient	Peak a: 0.96 MHz/°C		Peak c: 1.25 MHz/°C	
	Strain coefficient	Peak a: 0.048 MHz/με		Peak c: 0.055 MHz/με	
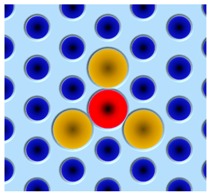 Ge-doped core and a triangularly-arranged F-doped buffer [135]	Core diameter	2.1 μm			
	Effective area	6.2 μm^2^			
	BFS (at 1550 nm)	Peak (1–5): 9.735 GHz, 10.009 GHz, 10.290 GHz, 10.524 GHz, 10.856 GHz			
	Temperature or strain coefficient	0.99 MHz/°C		0.038 MHz/με	
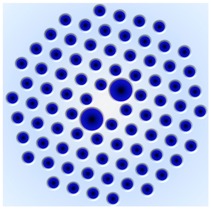 PM-PCF [136]	Core diameter	Large hole: 4.5 μm		Small hole: 2.2 μm	
	Mode field	Long axis: 3.6 μm		Short axis: 3.1 μm	
	Temperature coefficient	27.4 MHz/°C (−40~−15°C)	8 MHz/°C (−15~5°C)		Independent (5~80°C)
	Strain coefficient	−0.154 MHz/με			

**Table 10:** Brillouin scattering features of several PMFs.

Fiber Type	Fiber Parameters	Values	Application
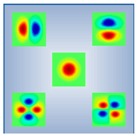 Five-mode [140]	Temperature coefficient	LP_01_: 1.01690 MHz/°C	Potential for simultaneous temperature and strain sensing
		LP_11_: 0.99099 MHz/°C	
	Strain coefficient	LP_01_: 0.05924 MHz/με	
		LP_11_: 0.07072MHz/με	
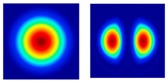 Ge-doped step-index FMF [141]	Temperature coefficient	LP_01_: 1.29 MHz/°C	
		LP_11_: 1.25 MHz/°C	
	Strain coefficient	LP_01_: 58.5 kHz/με	
		LP_11_: 57.6 kHz/με	
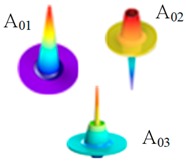 GI-FMF [142] ^1^	Temperature coefficient	A_01_: 5.27 MHz/°C	
		A_02_: 4.81 MHz/°C	
		A_03_: 4.30 MHz/°C	
	Strain coefficient	A_01_: 0.237 MHz/με	
		A_02_: 0.214 MHz/με	
		A_02_: 0.189 MHz/με	

^1^ The parameters were obtained based on finite-element method using COMSOL Multiphysics.

**Table 11:** Performance summary of EDF, LEAF and MCF.

Fiber Type	Fiber Parameters	Values	Application
Large effective-area fiber (LEAF) [145]	Temperature coefficient of the BBS power	Peak 1: 2.95 × 10^−3^/°C	Potential for simultaneous temperature and strain sensing
		Peak 2: 2.43 × 10^−3^/°C	
	Strain coefficient of the BBS power	Peak 1: −0.75× 10^−4^/με	
		Peak 2: −0.57× 10^−4^/με	
Erbium-doped fiber (EDF) [146,147]	BFS	11.42 GHz	
	Temperature coefficient	0.87 MHz/°C	
	Strain coefficient	479 MHz/%	
	Temperature coefficient of FIR	5.6 × 10^−4^/°C	
Multi-core fiber (MCF) [149].	BFS	Core 1: 10.73 GHz	
		Core 2: 10.85 GHz	
	Temperature coefficient	Core 1: 0.971 MHz/°C	
		Core 2: 0.959 MHz/°C	
	Strain coefficient	Core 1: 0.053 MHz/με	
		Core 2: 0.073 MHz/με	

## References

[1] Kapron F.P., Keck D.B., Maurer R.D. (1970). Radiation losses in glass optical waveguides. Appl. Phys. Lett..

[2] Kapron F., Maurer R., Teter M. (1972). Theory of backscattering effects in waveguides. Appl. Opt..

[3] Smith R.G. (1972). Optical power handling capacity of low loss optical fibers as determined by stimulated raman and brillouin scattering. Appl. Opt..

[4] Tosi D. (2017). Review and analysis of peak tracking techniques for fiber bragg grating sensors. Sensors.

[5] Personick S.D. (1977). Photon probe-an optical-fiber time-domain reflectometer. Bell Syst. Tech. J..

[6] Wang Y., Jin B.Q., Wang Y.C., Wang D., Liu X., Bai Q. (2017). Real-time distributed vibration monitoring system using phi-otdr. IEEE Sens. J..

[7] Liu X., Jin B.Q., Bai Q., Wang Y., Wang D., Wang Y.C. (2016). Distributed fiber-optic sensors for vibration detection. Sensors.

[8] Whitbread T.W., Wassef W.S., Allen P.M., Chu P.L. (1989). Profile dependence and measurement of absolute raman scattering cross-section in optical fibres. Electron. Lett..

[9] Culverhouse D., Farahi F., Pannell C., Jackson D. (1989). Potential of stimulated brillouin scattering as sensing mechanism for distributed temperature sensors. Electron. Lett..

[10] Horiguchi T., Kurashima T., Tateda M. (1989). Tensile strain dependence of brillouin frequency shift in silica optical fibers. IEEE Photonic Technol. Lett..

[11] Kurashima T., Horiguchi T., Tateda M. (1990). Distributed-temperature sensing using stimulated brillouin scattering in optical silica fibers. Opt. Lett..

[12] Kurashima T., Horiguchi T., Izumita H., Furukawa S., Koyamada Y. (1993). Brillouin optical-fiber time domain reflectometry. IEICE Trans. Commun..

[13] Garus D., Krebber K., Schliep F., Gogolla T. (1996). Distributed sensing technique based on brillouin optical-fiber frequency-domain analysis. Opt. Lett..

[14] Minardo A., Bernini R., Ruiz-Lombera R., Mirapeix J., Lopez-Higuera J.M., Zeni L. (2016). Proposal of brillouin optical frequency-domain reflectometry (bofdr). Opt. Express.

[15] Hotate K., Hasegawa T. (2000). Measurement of brillouin gain spectrum distribution along an optical fiber using a correlation-based technique—Proposal, experiment and simulation. IEICE Trans. Electron..

[16] Mizuno Y., Zou W., He Z., Hotate K. (2008). Proposal of brillouin optical correlation-domain reflectometry (bocdr). Opt. Express.

[17] Bao X., Chen L. (2011). Recent progress in brillouin scattering based fiber sensors. Sensors.

[18] Motil A., Bergman A., Tur M. (2016). State of the art of brillouin fiber-optic distributed sensing. Opt. Laser Technol..

[19] Pei H.-F., Teng J., Yin J.-H., Chen R. (2014). A review of previous studies on the applications of optical fiber sensors in geotechnical health monitoring. Measurement.

[20] Hong C., Zhang Y., Li G., Zhang M., Liu Z. (2017). Recent progress of using brillouin distributed fiber optic sensors for geotechnical health monitoring. Sens. Actuators A.

[21] Tosi D., Macchi E.G., Gallati M., Braschi G., Cigada A., Rossi S., Leen G., Lewis E. (2014). Fiber-optic chirped fbg for distributed thermal monitoring of ex-vivo radiofrequency ablation of liver. Biomed. Opt. Express.

[22] Tosi D., Schena E., Molardi C., Korganbayev S. (2018). Fiber optic sensors for sub-centimeter spatially resolved measurements: Review and biomedical applications. Opt. Fiber Technol..

[23] Zhang D., Xu H., Shi B., Sui H., Wei G. (2009). Brillouin power spectrum analysis for partially uniformly strained optical fiber. Opt. Laser. Eng..

[24] Dong Y., Zhang H., Chen L., Bao X. (2012). 2 cm spatial-resolution and 2 km range brillouin optical fiber sensor using a transient differential pulse pair. Appl. Opt..

[25] Diakaridia S., Pan Y., Xu P.B., Zhou D.W., Wang B.Z., Teng L., Lu Z.W., Ba D.X., Dong Y.K. (2017). Detecting cm-scale hot spot over 24-km-long single-mode fiber by using differential pulse pair botda based on double-peak spectrum. Opt. Express.

[26] Zhang J.Z., Feng C.K., Zhang M.J., Liu Y., Wu C.Y., Wang Y.H. (2018). Brillouin optical correlation domain analysis based on chaotic laser with suppressed time delay signature. Opt. Express.

[27] Bao X., Webb D.J., Jackson D.A. (1994). Combined distributed temperature and strain sensor based on brillouin loss in an optical fiber. Opt. Lett..

[28] Parker T.R., Farhadiroushan M., Handerek V.A., Rogers A.J. (1997). Temperature and strain dependence of the power level and frequency of spontaneous brillouin scattering in optical fibers. Opt. Lett..

[29] Kee H.H., Lees G.P., Newson T.P. (2000). All-fiber system for simultaneous interrogation of distributed strain and temperature sensing by spontaneous brillouin scattering. Opt. Lett..

[30] Bao X., Li W., Li Y., Chen L. Distributed fiber sensors based on stimulated brillouin scattering with centimeter spatial resolution. Proceedings of the International Conference on Optical Instruments and Technology—Microelectronic and Optoelectronic Devices and Integration.

[31] Naruse H., Tateda M. (1999). Trade-off between the spatial and the frequency resolutions in measuring the power spectrum of the brillouin backscattered light in an optical fiber. Appl. Opt..

[32] Wang F., Zhang X., Lu Y., Dou R., Bao X. (2009). Spatial resolution analysis for discrete fourier transform-based brillouin optical time domain reflectometry. Meas. Sci. Technol..

[33] Shibata R., Kasahara H., Elias L.P., Horiguchi T. (2017). Improving performance of phase shift pulse botdr. IEICE Electron. Express.

[34] Koyamada Y., Sakairi Y., Takeuchi N., Adachi S. (2007). Novel technique to improve spatial resolution in brillouin optical time-domain reflectometry. IEEE Photonic Technol. Lett..

[35] Sakairi Y., Matsuura S., Adachi S., Koyamada Y. Prototype double-pulse botdr for measuring distributed strain with 20-cm spatial resolution. Proceedings of the 2008 Annual Conference of the SICE.

[36] Nishiguchi K., Li C.H., Guzik A., Kishida K. (2014). Synthetic spectrum approach for brillouin optical time-domain reflectometry. Sensors.

[37] Shibata R., Kasahara H., Horiguchi T. Proposal and demonstration of high spatial resolution botdr by correlating signals sampled with narrow- and wide-width window functions. Proceedings of the 2016 IEEE 6th International Conference on Photonics.

[38] Zan M.S.D., Masui Y., Horiguchi T. Differential cross spectrum technique for improving the spatial resolution of botdr sensor. Proceedings of the IEEE 7th International Conference on Photonics (ICP).

[39] Li Q., Gan J., Wu Y., Zhang Z., Li J., Yang Z. (2016). High spatial resolution botdr based on differential brillouin spectrum technique. IEEE Photonic Technol. Lett..

[40] Yu Z., Zhang M., Dai H., Liu L., Zhang J., Jin X., Wang G. (2018). Distributed optical fiber sensing with brillouin optical time domain reflectometry based on differential pulse pair. Opt. Laser Technol..

[41] He J., Zhou Z., Ou J. (2013). Equidistance difference optimum method to enhance measuring space of brillouin optical fiber sensor. Opt. Eng..

[42] Wang F., Zhan W., Zhang X., Lu Y. (2013). Improvement of spatial resolution for botdr by iterative subdivision method. J. Lightwave Technol..

[43] Zhang Y., Li D., Fu X., Fu G., Jia W., Bi W. (2017). A pluse subdivision superposition method for improving the spatial resolution in botdr system. Optik.

[44] Yu Y.F., Luo L.Q., Li B., Soga K.C., Yan J.Z. (2017). Quadratic time-frequency transforms-based brillouin optical time-domain reflectometry. IEEE Sens. J..

[45] Feng X., Zhang X., Sun C., Motamedi M., Ansari F. (2013). Stationary wavelet transform method for distributed detection of damage by fiber-optic sensors. J. Eng. Mech..

[46] Hao Y., Ye Q., Pan Z., Cai H., Qu R. (2013). Influence of laser linewidth on performance of brillouin optical time domain reflectometry. Chin. Phys. B.

[47] Bai Q., Yan M., Xue B., Gao Y., Wang D., Wang Y., Zhang M., Zhang H., Jin B. (2019). The influence of laser linewidth on the brillouin shift frequency accuracy of botdr. Appl. Sci..

[48] Li C., Lu Y., Zhang X., Wang F. (2012). Snr enhancement in brillouin optical time domain reflectometer using multi-wavelength coherent detection. Electron. Lett..

[49] Lalam N., Ng W.P., Dai X., Wu Q., Fu Y.Q. Performance improvement of botdr system using wavelength diversity technique. Proceedings of the 25th International Conference on Optical Fiber Sensors.

[50] Lalam N., Ng W.P., Dai X., Wu Q., Fu Y.Q. (2018). Performance analysis of brillouin optical time domain reflectometry (botdr) employing wavelength diversity and passive depolarizer techniques. Meas. Sci. Technol..

[51] Lalam N., Ng W.P., Dai X., Wu Q., Fu Y.Q. (2018). Performance improvement of brillouin ring laser based botdr system employing a wavelength diversity technique. J. Lightwave Technol..

[52] Zhang Z., Lu Y., Zhao Y. (2018). Channel capacity of wavelength division multiplexing-based brillouin optical time domain sensors. IEEE Photonics J..

[53] Hao Y., Ye Q., Pan Z., Cai H., Qu R., Yang Z. (2013). Effects of modulated pulse format on spontaneous brillouin scattering spectrum and botdr sensing system. Opt. Laser Technol..

[54] Hao Y., Ye Q., Pan Z., Cai H., Qu R. (2013). Analysis of spontaneous brillouin scattering spectrum for different modulated pulse shape. Optik.

[55] Lu Y., Yao Y., Zhao X., Wang F., Zhang X. (2013). Influence of non-perfect extinction ratio of electro-optic modulator on signal-to-noise ratio of botdr. Opt. Commun..

[56] Zhang Y., Wu X., Ying Z., Zhang X. (2014). Performance improvement for long-range botdr sensing system based on high extinction ratio modulator. Electron. Lett..

[57] Bai Q., Xue B., Gu H., Wang D., Wang Y., Zhang M., Jin B., Wang Y. (2019). Enhancing the snr of botdr by gain-switched modulation. IEEE Photonic Technol. Lett..

[58] Wan S., Xiong Y., He X. (2014). The theoretical analysis and design of coding botdr system with apd detector. IEEE Sens. J..

[59] Soto M.A., Bolognini G., Pasquale F.D. (2008). Analysis of optical pulse coding in spontaneous brillouin-based distributed temperature sensors. Opt. Express.

[60] Soto M.A., Sahu P.K., Bolognini G., Di Pasquale F. (2008). Brillouin-based distributed temperature sensor employing pulse coding. IEEE Sens. J..

[61] Fan Z., Zhang X., Hu J., Zhang Y. Design of fast pulse coding/decoding system for botdr. Proceedings of the 2012 Photonics Global Conference (PGC).

[62] Hao Y., Ye Q., Pan Z., Cai H., Qu R. (2014). Digital coherent detection research on brillouin optical time domain reflectometry with simplex pulse codes. Chin. Phys. B.

[63] Lu Y., Liang H., Zhang X., Wang F. Brillouin optical time-domain reflectometry based on hadamard sequence probe pulse. Proceedings of the 9th International Conference on Optical Communications and Networks (ICOCN 2010).

[64] Li Y., Wang J., Yang Z. A method for improving botdr system performance. Proceedings of the 2012 Symposium on Photonics and Optoelectronics.

[65] Wang F., Zhu C., Cao C., Zhang X. (2017). Enhancing the performance of botdr based on the combination of fft technique and complementary coding. Opt. Express.

[66] Zhao Y., Zhang Y.-n., Han B., Qin C., Wang Q. (2013). High sensitive botdr demodulation method by using slow-light in fiber grating. J. Lightwave Technol..

[67] Wang Q., Zhao Y., Han B., Zhang Y.-n., Wang P., Wang L. (2014). A novel brillouin optical time-domain reflectometer demodulating method based on a slow-light mach-zehnder interferometer. Instrum. Sci. Technnol..

[68] Wang F., Li C., Zhao X., Zhang X. (2012). Using a mach-zehnder-interference-based passive configuration to eliminate the polarization noise in brillouin optical time domain reflectometry. Appl. Opt..

[69] Cao Y.L., Ye Q., Pan Z.Q., Cai H.W., Qu R.H., Fang Z.J., Zhao H. Mitigation of polarization fading in botdr sensors by using optical pulses with orthogonal polarizations. Proceedings of the 23rd International Conference on Optical Fibre Sensors.

[70] Wang R., Zhou L., Zhang X. (2014). Performance of brillouin optical time domain reflectometer with erbium doped fiber amplifier. Optik.

[71] Song M., Xia Q., Feng K., Lu Y., Yin C. (2016). 100 km brillouin optical time-domain reflectometer based on unidirectionally pumped raman amplification. Opt. Quant. Electron..

[72] Xia L., Hu J., Zhao Q., Chen J., Wu P., Zhang X. (2016). A distributed brillouin temperature sensor using a single-photon detector. IEEE Sens. J..

[73] Xia H., Shangguan M., Shentu G., Wang C., Qiu J., Zheng M., Xie X., Dou X., Zhang Q., Pan J.-W. (2016). Brillouin optical time-domain reflectometry using up-conversion single-photon detector. Opt. Commun..

[74] Li Y., Li X., An Q., Zhang L. (2017). Detrimental effect elimination of laser frequency instability in brillouin optical time domain reflectometer by using self-heterodyne detection. Sensors.

[75] Zhang Y.J., Li D., Fu X.H., Bi W.H. (2013). An improved levenberg-marquardt algorithm for extracting the features of brillouin scattering spectrum. Meas. Sci. Technol..

[76] Zhang Y., Zhao Y., Fu X., Xu J. (2016). A feature extraction method of the particle swarm optimization algorithm based on adaptive inertia weight and chaos optimization for brillouin scattering spectra. Opt. Commun..

[77] Liu R., Zhang M., Zhang J., Liu Y., Jin B., Bai Q., Li Z. (2016). Temperature measurement accuracy enhancement in the brillouin optical time domain reflectometry system using the sideband of brillouin gain spectrum demodulation. Acta Phys. Sin..

[78] Zhao L., Xu Z., Li Y. (2015). An accurate and rapid method for extracting parameters from multi-peak brillouin scattering spectra. Sens. Actuators A Phys..

[79] Soto M.A., Thevenaz L. (2013). Modeling and evaluating the performance of brillouin distributed optical fiber sensors. Opt. Express.

[80] Yu Y.F., Luo L.Q., Li B., Soga K., Yan J.Z. (2016). Frequency resolution quantification of brillouin-distributed optical fiber sensors. IEEE Photonic Technol. Lett..

[81] Zheng H., Fang Z., Wang Z., Lu B., Cao Y., Ye Q., Qu R., Cai H. (2018). Brillouin frequency shift of fiber distributed sensors extracted from noisy signals by quadratic fitting. Sensors.

[82] Yao Y., Lu Y., Zhang X., Wang F., Wang R. (2012). Reducing trade-off between spatial resolution and frequency accuracy in botdr using cohen’s class signal processing method. IEEE Photonic Technol. Lett..

[83] Huang M., Li W., Liu Z., Cheng L., Guan B.-O. (2018). Brillouin scattering spectrum analysis based on auto-regressive spectral estimation. Photonic Sens..

[84] Pradhan H.S., Sahu P.K. (2016). Brillouin distributed strain sensor performance improvement using fourward algorithm. Optik.

[85] Soto G., Fontbona J., Cortez R., Mujica L. (2016). An online two-stage adaptive algorithm for strain profile estimation from noisy and abruptly changing botdr data and application to underground mines. Measurement.

[86] Zhou D.W., Dong Y.K., Wang B.Z., Pang C., Ba D.X., Zhang H.Y., Lu Z.W., Li H., Bao X.Y. (2018). Single-shot botda based on an optical chirp chain probe wave for distributed ultrafast measurement. Light Sci. Appl..

[87] Zhang H.Y., Zhou D.W., Wang B.Z., Pang C., Xu P.B., Jiang T.F., Ba D.X., Li H., Dong Y.K. (2018). Recent progress in fast distributed brillouin optical fiber sensing. Appl. Sci..

[88] Ba D.X., Zhou D.W., Wang B.Z., Lu Z.W., Fan Z.G., Dong Y.K., Li H. (2017). Dynamic distributed brillouin optical fiber sensing based on dual-modulation by combining single frequency modulation and frequency-agility modulation. IEEE Photonics J..

[89] Ding Y., Shi B., Zhang D. (2010). Data processing in botdr distributed strain measurement based on pattern recognition. Optik.

[90] Wan S., He X., Fang L. (2012). Distributed brillouin fiber sensing based on spectrum line fitting and wavelet packet denoising. Opt. Commun..

[91] Yang W., Yang Y., Yang M. (2014). Fast digital envelope detector based on generalized harmonic wavelet transform for botdr performance improvement. Meas. Sci. Technol..

[92] Song M.P., Zhao B. (2005). Accuracy enhancement in brillouin scattering distributed temperature sensor based on hilbert transform. Opt. Commun..

[93] Wang X., Huang C.-X., Zhou L. Application of morlet wavelet in the extraction of brillouin scattering signal envelope. Proceedings of the 5th International Symposium on Photoelectronic Detection and Imaging (ISPDI)—Fiber Optic Sensors and Optical Coherence Tomography.

[94] Wang F., Zhan W., Lu Y., Yan Z., Zhang X. (2015). Determining the change of brillouin frequency shift by using the similarity matching method. J. Lightwave Technol..

[95] Abbasnejad M., Alizadeh B. (2018). Fpga-based implementation of a novel method for estimating the brillouin frequency shift in botda and botdr sensors. IEEE Sens. J..

[96] Geng J., Staines S., Blake M., Jiang S. (2007). Distributed fiber temperature and strain sensor using coherent radio-frequency detection of spontaneous brillouin scattering. Appl. Opt..

[97] Tu G., Zhang X., Zhang Y., Ying Z., Lv L. (2014). Strain variation measurement with short-time fourier transform-based brillouin optical time-domain reflectometry sensing system. Electron. Lett..

[98] Li B., Luo L., Yu Y., Soga K., Yan J. (2017). Dynamic strain measurement using small gain stimulated brillouin scattering in stft-botdr. IEEE Sens. J..

[99] Masoudi A., Belal M., Newson T.P. (2013). Distributed dynamic large strain optical fiber sensor based on the detection of spontaneous brillouin scattering. Opt. Lett..

[100] Koizumi K., Kanda Y., Fujii A., Murai H. High-speed distributed strain measurement using brillouin optical time-domain reflectometry based-on self-delayed heterodyne detection. Proceedings of the 2015 European Conference on Optical Communication (ECOC).

[101] Peled Y., Motil A., Yaron L., Tur M. (2011). Slope-assisted fast distributed sensing in optical fibers with arbitrary brillouin profile. Opt. Express.

[102] Zhou D., Dong Y., Wang B., Jiang T., Ba D., Xu P., Zhang H., Lu Z., Li H. (2017). Slope-assisted botda based on vector sbs and frequency-agile technique for wide-strain-range dynamic measurements. Opt. Express.

[103] Maraval D., Gabet R., Jaouen Y., Lamour V. (2017). Dynamic optical fiber sensing with brillouin optical time domain reflectometry: Application to pipeline vibration monitoring. J. Lightwave Technol..

[104] Shangguan M., Wang C., Xia H., Shentu G., Dou X., Zhang Q., Pan J.-w. (2017). Brillouin optical time domain reflectometry for fast detection of dynamic strain incorporating double-edge technique. Opt. Commun..

[105] Alahbabi M., Cho Y.T., Newson T.P. (2004). Comparison of the methods for discriminating temperature and strain in spontaneous brillouin-based distributed sensors. Opt. Lett..

[106] Ba D., Chen C., Fu C., Zhang D., Lu Z., Fan Z., Dong Y. (2018). A high-performance and temperature-insensitive shape sensor based on dpp-botda. IEEE Photonics J..

[107] Parker T.R., Farhadiroushan M., Handerek V.A., Roger A.J. (1997). A fully distributed simultaneous strain and temperature sensor using spontaneous brillouin backscatter. IEEE Photonic Technol. Lett..

[108] Alahbabi M.N., Cho Y.T., Newson T.P. (2005). Simultaneous temperature and strain measurement with combined spontaneous raman and brillouin scattering. Opt. Lett..

[109] Bolognini G., Soto M.A., Di Pasquale F. (2009). Fiber-optic distributed sensor based on hybrid raman and brillouin scattering employing multiwavelength fabry-perot lasers. IEEE Photonic Technol. Lett..

[110] Xia H., Zhang C., Mu H., Sun D. (2009). Edge technique for direct detection of strain and temperature based on optical time domain reflectometry. Appl. Opt..

[111] Soto M.A., Bolognini G., Di Pasquale F. (2009). Enhanced simultaneous distributed strain and temperature fiber sensor employing spontaneous brillouin scattering and optical pulse coding. IEEE Photonic Technol. Lett..

[112] Taki M., Signorini A., Oton C.J., Nannipieri T., Di Pasquale F. (2013). Hybrid raman/brillouin-optical-time-domain-analysis-distributed optical fiber sensors based on cyclic pulse coding. Opt. Lett..

[113] Kim S., Kwon H., Yang I., Lee S., Kim J., Kang S. (2013). Performance of a distributed simultaneous strain and temperature sensor based on a fabry-perot laser diode and a dual-stage fbg optical demultiplexer. Sensors.

[114] Yu Y., Luo L., Li B., Guo L., Yan J., Soga K. (2015). Double peak-induced distance error in short-time-fourier-transform-brillouin optical time domain reflectometers event detection and the recovery method. Appl. Opt..

[115] Cui Q., Pamukcu S., Xiao W., Guintrand C., Toulouse J., Pervizpour M. (2009). Distributed fiber sensor based on modulated pulse base reflection and brillouin gain spectrum analysis. Appl. Opt..

[116] Breteler R.F.K., van der Tol J.J.G.M., Felicetti M., Sasbrink B., Smit M.K. (2011). Photonic integrated brillouin optical time domain reflection readout unit. Opt. Eng..

[117] Lida D., Ito F. (2009). Cost-effective bandwidth-reduced brillouin optical time domain reflectometry using a reference brillouin scattering beam. Appl. Opt..

[118] Hao Y., Ye Q., Pan Z., Yang F., Cai H., Qu R., Zhang Q., Yang Z. (2012). Design of wide-band frequency shift technology by using compact brillouin fiber laser for brillouin optical time domain reflectometry sensing system. IEEE Photonics J..

[119] Bai Q., Zheng X., Wang D., Wang Y., Liu X., Zhang M.J., Zhang H.J., Jin B.Q. (2018). A logarithmic detection scheme in botdr with low-bandwidth requests. IEEE Access.

[120] Wang F., Zhang X., Wang X., Chen H. (2013). Distributed fiber strain and vibration sensor based on brillouin optical time-domain reflectometry and polarization optical time-domain reflectometry. Opt. Lett..

[121] Zhang J., Zhu T., Zhou H., Huang S., Liu M., Huang W. (2016). High spatial resolution distributed fiber system for multi-parameter sensing based on modulated pulses. Opt. Express.

[122] Husdi I.R., Nakamura K., Ueha S. (2004). Sensing characteristics of plastic optical fibres measured by optical time-domain reflectometry. Meas. Sci. Technol..

[123] Mizuno Y., Nakamura K. (2010). Experimental study of brillouin scattering in perfluorinated polymer optical fiber at telecommunication wavelength. Appl. Phys. Lett..

[124] Mizuno Y., Nakamura K. (2010). Potential of brillouin scattering in polymer optical fiber for strain-insensitive high-accuracy temperature sensing. Opt. Lett..

[125] Mizuno Y., Ishigure T., Nakamura K. (2011). Brillouin gain spectrum characterization in perfluorinated graded-index polymer optical fiber with 62.5-μm core diameter. IEEE Photonic Technol. Lett..

[126] Hayashi N., Mizuno Y., Nakamura K. (2012). Brillouin gain spectrum dependence on large strain in perfluorinated graded-index polymer optical fiber. Opt. Express.

[127] Minakawa K., Mizuno Y., Nakamura K. (2017). Cross effect of strain and temperature on brillouin frequency shift in polymer optical fibers. J. Lightwave Technol..

[128] Hayashi N., Mizuno Y., Koyama D., Nakamura K. (2012). Dependence of brillouin frequency shift on temperature and strain in poly(methyl methacrylate)-based polymer optical fibers estimated by acoustic velocity measurement. Appl. Phys. Express.

[129] Hayashi N., Mizuno Y., Koyama D., Nakamura K. (2011). Measurement of acoustic velocity in poly(methyl methacrylate)-based polymer optical fiber for brillouin frequency shift estimation. Appl. Phys. Express.

[130] Minakawa K., Koike K., Hayashi N., Koike Y., Mizuno Y., Nakamura K. (2016). Dependence of brillouin frequency shift on water absorption ratio in polymer optical fibers. J. Appl. Phys..

[131] Minakawa K., Hayashi N., Mizuno Y., Nakamura K. (2013). Potential applicability of brillouin scattering in partially chlorinated polymer optical fibers to high-precision temperature sensing. Appl. Phys. Express.

[132] Kawa T., Numata G., Lee H., Hayashi N., Mizuno Y., Nakamura K. (2017). Single-end-access strain and temperature sensing based on multimodal interference in polymer optical fibers. IEICE Electron. Express.

[133] Zou L., Bao X., Chen L. (2003). Brillouin scattering spectrum in photonic crystal fiber with a partially germanium-doped core. Opt. Lett..

[134] Zou L., Bao X., Afshar S., Chen L. (2004). Dependence of the brillouin frequency shift on strain and temperature in a photonic crystal fiber. Opt. Lett..

[135] Zou W., He Z., Hotate K. (2012). Experimental investigation on brillouin scattering property in highly nonlinear photonic crystal fiber with hybrid core. Opt. Express.

[136] Zhang H., Yuan Z., Liu Z., Gao W., Dong Y. (2017). Simultaneous measurement of strain and temperature using a polarization-maintaining photonic crystal fiber with stimulated brillouin scattering. Appl. Phys. Express.

[137] Song K.Y., Kim Y.H. (2013). Characterization of stimulated brillouin scattering in a few-mode fiber. Opt. Lett..

[138] Song K.Y., Kim Y.H., Kim B.Y. (2013). Intermodal stimulated brillouin scattering in two-mode fibers. Opt. Lett..

[139] Li A., Wang Y.F., Hu Q., Che D., Chen X., Shieh W. (2014). Measurement of distributed mode coupling in a few-mode fiber using a reconfigurable brillouin otdr. Opt. Lett..

[140] Li A., Wang Y., Fang J., Li M.J., Kim B.Y., Shieh W. (2015). Few-mode fiber multi-parameter sensor with distributed temperature and strain discrimination. Opt. Lett..

[141] Weng Y., Ip E., Pan Z., Wang T. (2015). Single-end simultaneous temperature and strain sensing techniques based on brillouin optical time domain reflectometry in few-mode fibers. Opt. Express.

[142] Zhou X., Guo Z., Ke C., Liu D. Simultaneous temperature and strain sensing utilizing brillouin frequency shifts contributed by multiple acoustic modes. Proceedings of the 29th IEEE Photonics Conference (IPC).

[143] Wu H., Wang R., Liu D., Fu S., Zhao C., Wei H., Tong W., Shum P.P., Tang M. (2016). Few-mode fiber based distributed curvature sensor through quasi-single-mode brillouin frequency shift. Opt. Lett..

[144] Liu X., Bao X.Y. (2012). Brillouin spectrum in leaf and simultaneous temperature and strain measurement. J. Lightwave Technol..

[145] Lu Y., Qin Z., Lu P., Zhou D., Chen L., Bao X. (2013). Distributed strain and temperature measurement by brillouin beat spectrum. IEEE Photonic Technol. Lett..

[146] Ding M.J., Hayashi N., Mizuno Y., Nakamura K. (2013). Brillouin gain spectrum dependences on temperature and strain in erbium-doped optical fibers with different erbium concentrations. Appl. Phys. Lett..

[147] Ding M.J., Mizuno Y., Nakamura K. (2014). Discriminative strain and temperature measurement using brillouin scattering and fluorescence in erbium-doped optical fiber. Opt. Express.

[148] Zhao Z., Dang Y., Tang M., Duan L., Wang M., Wu H., Fu S., Tong W., Shum P.P., Liu D. (2016). Spatial-division multiplexed hybrid raman and brillouin optical time-domain reflectometry based on multi-core fiber. Opt. Express.

[149] Zaghloul M.A.S., Wang M., Milione G., Li M.J., Li S.P., Huang Y.K., Wang T., Chen K.P. (2018). Discrimination of temperature and strain in brillouin optical time domain analysis using a multicore optical fiber. Sensors.

[150] Luo L., Parmigiani F., Yu Y., Li B., Soga K., Yan J. (2018). Frequency uncertainty improvement in a stft-botdr using highly nonlinear optical fibers. Opt. Express.

[151] Lanticq V., Bourgeois E., Magnien P., Dieleman L., Vinceslas G., Sang A., Delepine-Lesoille S. (2009). Soil-embedded optical fiber sensing cable interrogated by brillouin optical time-domain reflectometry (b-otdr) and optical frequency-domain reflectometry (ofdr) for embedded cavity detection and sinkhole warning system. Meas. Sci. Technol..

[152] Klar A., Linker R. (2010). Feasibility study of automated detection of tunnel excavation by brillouin optical time domain reflectometry. Tunn. Undergr. Space Technol..

[153] Mohamad H., Soga K., Bennett P.J., Mair R.J., Lim C.S. (2012). Monitoring twin tunnel interaction using distributed optical fiber strain measurements. J. Geotech. Geoenviron. Eng..

[154] Webb G.T., Vardanega P.J., Hoult N.A., Fidler P.R.A., Bennett P.J., Middleton C.R. (2017). Analysis of fiber-optic strain-monitoring data from a prestressed concrete bridge. J. Bridge Eng..

[155] He J., Zhou Z., Jinping O. (2013). Optic fiber sensor-based smart bridge cable with functionality of self-sensing. Mech. Syst. Signal. Process..

[156] Liu W.Q., Wang H.P., Zhou Z., Li S.Y., Ni Y.B., Wang G. Optical fiber based sensing system design for the health monitoring of multi-layered pavement structure. Proceedings of the International Conference on Optical Instruments and Technology (OIT)-Optical Sensors and Applications.

[157] Yan S.Z., Chyan L.S. (2010). Performance enhancement of botdr fiber optic sensor for oil and gas pipeline monitoring. Opt. Fiber Technol..

[158] Mirzaei A., Bahrampour A.R., Taraz M., Bahrampour A., Bahrampour M.J., Ahmadi Foroushani S.M. (2013). Transient response of buried oil pipelines fiber optic leak detector based on the distributed temperature measurement. Int. J. Heat Mass Transf..

[159] Madabhushi S.S.C., Elshafie M.Z.E.B., Haigh S.K. (2015). Accuracy of distributed optical fiber temperature sensing for use in leak detection of subsea pipelines. J. Pipeline Syst. Eng. Pract..

[160] Feng X., Wu W., Li X., Zhang X., Zhou J. (2015). Experimental investigations on detecting lateral buckling for subsea pipelines with distributed fiber optic sensors. Smart Struct. Syst..

[161] Bai Q., Yan W., Wang D., Wang Y., Liu X., Jin B.Q. Multi-parameter cbm pipeline safety monitoring system based on optical fiber sensing. Proceedings of the International Symposium on Optoelectronic Technology and Application (OTA).

[162] Lu Y., Shi B., Wei G.Q., Chen S.E., Zhang D. (2012). Application of a distributed optical fiber sensing technique in monitoring the stress of precast piles. Smart Mater. Struct..

[163] Sun Y., Shi B., Chen S.-E., Zhu H., Zhang D., Lu Y. (2014). Feasibility study on corrosion monitoring of a concrete column with central rebar using botdr. Smart Struct. Syst..

[164] Feng S.J., Lu S.F., Shi Z.M. (2015). Field investigations of two super-long steel pipe piles in offshore areas. Mar. Georesour. Geotechnol..

[165] Nan S., Gao Q. Application of distributed optical fiber sensor technology based on botdr in similar model test of backfill mining. Proceedings of the 2nd International Conference on Mining Engineering and Metallurgical Technology.

[166] Wang S., Luan L. Analysis on the security monitoring and detection of mine roof collapse based on botdr technology. Proceedings of the 2nd International Conference on Soft Computing in Information Communication Technology (SCICT).

[167] Moffat R., Sotomayor J., Beltrán J.F. (2015). Estimating tunnel wall displacements using a simple sensor based on a brillouin optical time domain reflectometer apparatus. Int. J. Rock Mech. Min. Sci..

[168] Wang B.-J., Li K., Shi B., Wei G.-Q. (2009). Test on application of distributed fiber optic sensing technique into soil slope monitoring. Landslides.

[169] Yin Y., Wang H., Gao Y., Li X. (2010). Real-time monitoring and early warning of landslides at relocated wushan town, the three gorges reservoir, china. Landslides.

[170] Gu K., Shi B., Liu C., Jiang H., Li T., Wu J. (2018). Investigation of land subsidence with the combination of distributed fiber optics sensing techniques and microstructure analysis of soils. Eng. Geol..

[171] Klar A., Dromy I., Linker R. (2014). Monitoring tunneling induced ground displacements using distributed fiber-optic sensing. Tunn. Undergr. Space Technol..

[172] Luo J., Hao Y., Ye Q., Hao Y., Li L. (2013). Development of optical fiber sensors based on brillouin scattering and fbg for on-line monitoring in overhead transmission lines. J. Lightwave Technol..

[173] Lv A.Q., Li Y.Q., Li J. Research on strain and temperature measurement of opgw based on botdr. Proceedings of the International Conference on Optical Instruments and Technology (OIT)—Optical Sensors and Applications.

[174] Lu L.D., Liang Y., Li B.L., Guo J.H., Zhang H., Zhang X.P. Health monitoring of electric power communication line using a distributed optical fiber sensor. Proceedings of the Conference on Advanced Sensor Systems and Applications VI.

[175] Zhao L., Li Y., Xu Z., Yang Z., Lü A. (2014). On-line monitoring system of 110kv submarine cable based on botdr. Sensor Actuators A Phys..

[176] Hao Y., Cao Y., Ye Q., Cai H., Qu R. (2015). On-line temperature monitoring in power transmission lines based on brillouin optical time domain reflectometry. Optik.

[177] Zhou Z., Huang M., He J., Chen G., Ou J. (2010). Ice structure monitoring with an optical fiber sensing system. Cold Reg. Sci. Technol..

[178] Gao L., Shi B., Zhu Y., Wang K., Sun Y., Tang C. (2011). A distributed soil temperature measurement system with high spatial resolution based on botdr. Opt. Appl..

[179] Luo L., Li B., Yu Y., Xu X., Soga K., Yan J. (2016). Time and frequency localized pulse shape for resolution enhancement in stft-botdr. J. Sens..

[180] Ruiz-Lombera R., Fuentes A., Rodriguez-Cobo L., Miguel Lopez-Higuera J., Mirapeix J. (2018). Simultaneous temperature and strain discrimination in a conventional botda via artificial neural networks. J. Lightwave Technol..

[181] Azad A.K., Wang L., Guo N., Lu C., Tam H.Y. (2015). Temperature sensing in botda system by using artificial neural network. Electron. Lett..

[182] Azad A.K., Wang L., Guo N., Tam H.Y., Lu C. (2016). Signal processing using artificial neural network for botda sensor system. Opt. Express.

[183] Wu H., Wang L., Guo N., Shu C., Lu C. (2017). Support vector machine assisted botda utilizing combined brillouin gain and phase information for enhanced sensing accuracy. Opt. Express.

[184] Wu H., Wang L., Guo N., Shu C., Lu C. (2017). Brillouin optical time-domain analyzer assisted by support vector machine for ultrafast temperature extraction. J. Lightwave Technol..

[185] Wu H., Wang L., Zhao Z., Shu C., Lu C. (2018). Support vector machine based differential pulse-width pair brillouin optical time domain analyzer. IEEE Photonics J..

[186] Wang B., Guo N., Wang L., Yu C., Lu C. Denoising and robust temperature extraction for botda systems based on denoising autoencoder and dnn. Proceedings of the 26th International Conference on Optical Fiber Sensors.

[187] Fu Y., Wang Z., Zhu R., Xue N., Jiang J., Lu C., Zhang B., Yang L., Atubga D., Rao Y. (2018). Ultra-long-distance hybrid botda/phi-otdr. Sensors.

[188] Zhang X., Hu J., Zhang Y. (2013). A hybrid single-end-access botda and cotdr sensing system using heterodyne detection. J. Lightwave Technol..

